# Autoantibodies against type I IFNs in patients with life-threatening COVID-19

**DOI:** 10.1126/science.abd4585

**Published:** 2020-09-24

**Authors:** Paul Bastard, Lindsey B. Rosen, Qian Zhang, Eleftherios Michailidis, Hans-Heinrich Hoffmann, Yu Zhang, Karim Dorgham, Quentin Philippot, Jérémie Rosain, Vivien Béziat, Jérémy Manry, Elana Shaw, Liis Haljasmägi, Pärt Peterson, Lazaro Lorenzo, Lucy Bizien, Sophie Trouillet-Assant, Kerry Dobbs, Adriana Almeida de Jesus, Alexandre Belot, Anne Kallaste, Emilie Catherinot, Yacine Tandjaoui-Lambiotte, Jeremie Le Pen, Gaspard Kerner, Benedetta Bigio, Yoann Seeleuthner, Rui Yang, Alexandre Bolze, András N. Spaan, Ottavia M. Delmonte, Michael S. Abers, Alessandro Aiuti, Giorgio Casari, Vito Lampasona, Lorenzo Piemonti, Fabio Ciceri, Kaya Bilguvar, Richard P. Lifton, Marc Vasse, David M. Smadja, Mélanie Migaud, Jérome Hadjadj, Benjamin Terrier, Darragh Duffy, Lluis Quintana-Murci, Diederik van de Beek, Lucie Roussel, Donald C. Vinh, Stuart G. Tangye, Filomeen Haerynck, David Dalmau, Javier Martinez-Picado, Petter Brodin, Michel C. Nussenzweig, Stéphanie Boisson-Dupuis, Carlos Rodríguez-Gallego, Guillaume Vogt, Trine H. Mogensen, Andrew J. Oler, Jingwen Gu, Peter D. Burbelo, Jeffrey I. Cohen, Andrea Biondi, Laura Rachele Bettini, Mariella D'Angio, Paolo Bonfanti, Patrick Rossignol, Julien Mayaux, Frédéric Rieux-Laucat, Eystein S. Husebye, Francesca Fusco, Matilde Valeria Ursini, Luisa Imberti, Alessandra Sottini, Simone Paghera, Eugenia Quiros-Roldan, Camillo Rossi, Riccardo Castagnoli, Daniela Montagna, Amelia Licari, Gian Luigi Marseglia, Xavier Duval, Jade Ghosn, HGID Lab, NIAID-USUHS Immune Response to COVID Group, COVID Clinicians, COVID-STORM Clinicians, Imagine COVID Group, French COVID Cohort Study Group, The Milieu Intérieur Consortium, CoV-Contact Cohort, Amsterdam UMC Covid-19 Biobank, COVID Human Genetic Effort, John S. Tsang, Raphaela Goldbach-Mansky, Kai Kisand, Michail S. Lionakis, Anne Puel, Shen-Ying Zhang, Steven M. Holland, Guy Gorochov, Emmanuelle Jouanguy, Charles M. Rice, Aurélie Cobat, Luigi D. Notarangelo, Laurent Abel, Helen C. Su, Jean-Laurent Casanova

**Affiliations:** 1 Laboratory of Human Genetics of Infectious Diseases, Necker Branch, INSERM U1163, Necker Hospital for Sick Children, Paris, France.; 2 University of Paris, Imagine Institute, Paris, France.; 3 St. Giles Laboratory of Human Genetics of Infectious Diseases, Rockefeller Branch, The Rockefeller University, New York, NY, USA.; 4 Laboratory of Clinical Immunology and Microbiology, Division of Intramural Research, National Institute of Allergy and Infectious Diseases (NIAID), National Institutes of Health (NIH), Bethesda, MD, USA.; 5 Laboratory of Virology and Infectious Disease, The Rockefeller University, New York, NY, USA.; 6 Sorbonne Université, INSERM, Centre d’Immunologie et des Maladies Infectieuses, (CIMI-Paris), Paris, France.; 7 Institute of Biomedicine and Translational Medicine, University of Tartu, Tartu, Estonia.; 8 Hospices Civils de Lyon, Lyon Sud Hospital, Pierre-Bénite, France.; 9 International Center of Research in Infectiology, Lyon University, INSERM U1111, CNRS UMR 5308, ENS, UCBL, Lyon, France.; 10 International Center of Research in Infectiology, Lyon University, INSERM U1111, CNRS UMR 5308, ENS, UCBL, Lyon, France.; 11 National Referee Centre for Rheumatic and AutoImmune and Systemic Diseases in Children (RAISE), Lyon, France.; 12 Lyon Immunopathology Federation (LIFE), Hospices Civils de Lyon, Lyon, France.; 13 Internal Medicine Clinic, Tartu University Hospital, Tartu, Estonia.; 14 Pneumology Department, Foch Hospital, Suresne, France.; 15 Avicenne Hospital, Assistance Publique Hôpitaux de Paris (AP-HP), Bobigny, INSERM U1272 Hypoxia and Lung, Bobigny, France.; 16 Helix, San Mateo, CA, USA.; 17 Department of Medical Microbiology, University Medical Center Utrecht, Utrecht, Netherlands.; 18 IRCCS San Raffaele Hospital and Vita-Salute San Raffaele University, Milan, Italy.; 19 Department of Genetics, Yale University School of Medicine, New Haven, CT, USA.; 20 Yale Center for Genome Analysis, Yale University School of Medicine, New Haven, CT, USA.; 21 Laboratory of Human Genetics and Genomics, The Rockefeller University, New York, NY, USA.; 22 Service de Biologie Clinique and UMR-S 1176, Hôpital Foch, Suresnes, France.; 23 INSERM UMR-S 1140, Biosurgical Research Laboratory (Carpentier Foundation), Paris University and European Georges Pompidou Hospital, Paris, France.; 24 Laboratory of Immunogenetics of Pediatric Autoimmune Diseases, INSERM UMR 1163, University of Paris, Imagine Institute, Paris, France.; 25 Department of Internal Medicine, National Referral Center for Rare Systemic Autoimmune Diseases, Assistance Publique Hôpitaux de Paris-Centre (APHP-CUP), University of Paris, Paris, France.; 26 Translational Immunology Laboratory, Institut Pasteur, Paris, France.; 27 Human Evolutionary Genetics Unit, Institut Pasteur, CNRS UMR 2000, 75015, Paris, France.; 28 Human Genomics and Evolution, Collège de France, Paris, France.; 29 Amsterdam UMC, University of Amsterdam, Department of Neurology, Amsterdam Neuroscience, Amsterdam, Netherlands.; 30 Department of Medicine, Division of Infectious Diseases, McGill University Health Centre, Montréal, Québec, Canada.; 31 Infectious Disease Susceptibility Program, Research Institute, McGill University Health Centre, Montréal, Québec, Canada.; 32 Garvan Institute of Medical Research, Darlinghurst 2010, NSW, Sydney, Australia.; 33 St Vincent’s Clinical School, Faculty of Medicine, University of New South Wales Sydney, Darlinghurst 2010, NSW, Australia.; 34 Department of Paediatric Immunology and Pulmonology, Centre for Primary Immunodeficiency Ghent (CPIG), PID Research Laboratory, Jeffrey Modell Diagnosis and Research Centre, Ghent University Hospital, Ghent, Belgium.; 35 Infectious Diseases and HIV Service, Hospital Universitari Mutua Terrassa, Universitat de Barcelona, Fundació Docència i Recerca Mutua Terrassa, Terrassa, Barcelona, Catalonia, Spain.; 36 IrsiCaixa AIDS Research Institute and Institute for Health Science Research Germans Trias i Pujol (IGTP), Badalona, Spain.; 37 Infectious Diseases and Immunity, Centre for Health and Social Care Research (CESS), Faculty of Medicine, University of Vic-Central University of Catalonia (UVic-UCC), Vic, Spain.; 38 Catalan Institution for Research and Advanced Studies (ICREA), Barcelona, Spain.; 39 Science for Life Laboratory, Department of Women's and Children's Health, Karolinska Institutet, Karolinska, Sweden.; 40 Department of Pediatric Rheumatology, Karolinska University Hospital, Karolinska, Sweden.; 41 Laboratory of Molecular Immunology, The Rockefeller University, New York, NY, USA.; 42 Howard Hughes Medical Institute, New York, NY, USA.; 43 Department of Immunology, Hospital Universitario de Gran Canaria Dr. Negrín, Canarian Health System, Las Palmas de Gran Canaria, Spain.; 44 Department of Clinical Sciences, University Fernando Pessoa Canarias, Las Palmas de Gran Canaria, Spain.; 45 Neglected Human Genetics Laboratory, INSERM, University of Paris, Paris, France.; 46 Department of Infectious Diseases, Aarhus University Hospital, Skejby, Denmark.; 47 Department of Biomedicine, Aarhus University, Aarhus, Denmark.; 48 Bioinformatics and Computational Biosciences Branch, Office of Cyber Infrastructure and Computational Biology, NIAID, NIH, Bethesda, MD, USA.; 49 Division of Intramural Research, National Institute of Dental Craniofacial Research (NIDCR), NIH, Bethesda, MD, USA.; 50 Laboratory of Infectious Diseases, Division of Intramural Research, NIAID, NIH, Bethesda, MD, USA.; 51 Pediatric Department and Centro Tettamanti-European Reference Network PaedCan, EuroBloodNet, MetabERN-University of Milano-Bicocca-Fondazione MBBM-Ospedale, San Gerardo, Monza, Italy.; 52 Department of Infectious Diseases, San Gerardo Hospital - University of Milano-Bicocca, Monza, Italy.; 53 University of Lorraine, Plurithematic Clinical Investigation Centre INSERM CIC-P 1433, INSERM U1116, CHRU Nancy Hopitaux de Brabois, F-CRIN INI-CRCT (Cardiovascular and Renal Clinical Trialists), Nancy, France.; 54 Intensive Care Unit, Pitié-Salpétrière Hospital, Paris University, AP-HP, Paris, France.; 55 Department of Clinical Science and K.G. Jebsen Center for Autoimmune Disorders, University of Bergen, Bergen, Norway.; 56 Department of Medicine, Haukeland University Hospital, Bergen, Norway.; 57 Department of Medicine (Solna), Karolinska Institutet, Stockholm, Sweden.; 58 Human Molecular Genetics Laboratory, Institute of Genetics and Biophysics, “A. Buzzati-Traverso” Consiglio Nazionale delle Ricerche, Naples, Italy.; 59 Centro di Ricerca Emato-oncologica AIL (CREA) Laboratory, Diagnostic Department, ASST Spedali Civili di Brescia, Brescia, Italy.; 60 Department of Infectious and Tropical Diseases, University of Brescia and ASST Spedali di Brescia, Brescia, Italy.; 61 Direzione Sanitaria, ASST Spedali Civili di Brescia, Brescia, Italy.; 62 Department of Pediatrics, Fondazione IRCCS Policlinico San Matteo, University of Pavia, Pavia, Italy.; 63 Laboratory of Immunology and Transplantation, Fondazione IRCCS Policlinico San Matteo, Pavia, Italy.; 64 Department of Clinical, Surgical, Diagnostic and Pediatric Sciences, University of Pavia, Pavia, Italy.; 65 INSERM CIC 1425, Paris, France.; 66 AP-HP, University Hospital of Bichat, Paris, France.; 67 University Paris Diderot, Paris 7, UFR de Médecine-Bichat, Paris, France.; 68 Infection, Antimicrobials, Modelling, Evolution (IAME), INSERM, UMRS1137, University of Paris, Paris, France.; 69 AP-HP, Bichat Claude Bernard Hospital, Infectious and Tropical Diseases Department, Paris, France.; 70 Center for Human Immunology, NIH, Bethesda, MD, USA.; 71 Multiscale Systems Biology Section, Laboratory of Immune System Biology, NIAID, NIH, Bethesda, MD, USA.; 72 Département d’Immunologie, AP-HP, Hôpital Pitié-Salpétrière, Paris, France.; 73 Pediatric Hematology and Immunology Unit, Necker Hospital for Sick Children, AP-HP, Paris, France.

## Abstract

Interindividual clinical variability in the course of severe acute respiratory syndrome coronavirus 2 (SARS-CoV-2) infection is vast. We report that at least 101 of 987 patients with life-threatening coronavirus disease 2019 (COVID-19) pneumonia had neutralizing immunoglobulin G (IgG) autoantibodies (auto-Abs) against interferon-ω (IFN-ω) (13 patients), against the 13 types of IFN-α (36), or against both (52) at the onset of critical disease; a few also had auto-Abs against the other three type I IFNs. The auto-Abs neutralize the ability of the corresponding type I IFNs to block SARS-CoV-2 infection in vitro. These auto-Abs were not found in 663 individuals with asymptomatic or mild SARS-CoV-2 infection and were present in only 4 of 1227 healthy individuals. Patients with auto-Abs were aged 25 to 87 years and 95 of the 101 were men. A B cell autoimmune phenocopy of inborn errors of type I IFN immunity accounts for life-threatening COVID-19 pneumonia in at least 2.6% of women and 12.5% of men.

## The genetics underlying severe COVID-19

The immune system is complex and involves many genes, including those that encode cytokines known as interferons (IFNs). Individuals that lack specific IFNs can be more susceptible to infectious diseases. Furthermore, the autoantibody system dampens IFN response to prevent damage from pathogen-induced inflammation. Two studies now examine the likelihood that genetics affects the risk of severe coronavirus disease 2019 (COVID-19) through components of this system (see the Perspective by Beck and Aksentijevich). Q. Zhang *et al.* used a candidate gene approach and identified patients with severe COVID-19 who have mutations in genes involved in the regulation of type I and III IFN immunity. They found enrichment of these genes in patients and conclude that genetics may determine the clinical course of the infection. Bastard *et al.* identified individuals with high titers of neutralizing autoantibodies against type I IFN-α2 and IFN-ω in about 10% of patients with severe COVID-19 pneumonia. These autoantibodies were not found either in infected people who were asymptomatic or had milder phenotype or in healthy individuals. Together, these studies identify a means by which individuals at highest risk of life-threatening COVID-19 can be identified.

*Science*, this issue p. eabd4570, p. eabd4585; see also p. 404

## Abstract (structured)

**INTRODUCTION:**

Interindividual clinical variability is vast in humans infected with severe acute respiratory syndrome coronavirus 2 (SARS-CoV-2), ranging from silent infection to rapid death. Three risk factors for life-threatening coronavirus disease 2019 (COVID-19) pneumonia have been identified—being male, being elderly, or having other medical conditions—but these risk factors cannot explain why critical disease remains relatively rare in any given epidemiological group. Given the rising toll of the COVID-19 pandemic in terms of morbidity and mortality, understanding the causes and mechanisms of life-threatening COVID-19 is crucial.

**RATIONALE:**

B cell autoimmune infectious phenocopies of three inborn errors of cytokine immunity exist, in which neutralizing autoantibodies (auto-Abs) against interferon-γ (IFN-γ) (mycobacterial disease), interleukin-6 (IL-6) (staphylococcal disease), and IL-17A and IL-17F (mucocutaneous candidiasis) mimic the clinical phenotypes of germline mutations of the genes that encode the corresponding cytokines or receptors. Human inborn errors of type I IFNs underlie severe viral respiratory diseases. Neutralizing auto-Abs against type I IFNs, which have been found in patients with a few underlying noninfectious conditions, have not been unequivocally shown to underlie severe viral infections. While searching for inborn errors of type I IFN immunity in patients with life-threatening COVID-19 pneumonia, we also tested the hypothesis that neutralizing auto-Abs against type I IFNs may underlie critical COVID-19. We searched for auto-Abs against type I IFNs in 987 patients hospitalized for life-threatening COVID-19 pneumonia, 663 asymptomatic or mildly affected individuals infected with SARS-CoV-2, and 1227 healthy controls from whom samples were collected before the COVID-19 pandemic.

**RESULTS:**

At least 101 of 987 patients (10.2%) with life-threatening COVID-19 pneumonia had neutralizing immunoglobulin G (IgG) auto-Abs against IFN-ω (13 patients), against the 13 types of IFN-α (36), or against both (52) at the onset of critical disease; a few also had auto-Abs against the other three individual type I IFNs. These auto-Abs neutralize high concentrations of the corresponding type I IFNs, including their ability to block SARS-CoV-2 infection in vitro. Moreover, all of the patients tested had low or undetectable serum IFN-α levels during acute disease. These auto-Abs were present before infection in the patients tested and were absent from 663 individuals with asymptomatic or mild SARS-CoV-2 infection (*P* < 10^−16^). They were present in only 4 of 1227 (0.33%) healthy individuals (*P* < 10^−16^) before the pandemic. The patients with auto-Abs were 25 to 87 years old (half were over 65) and of various ancestries. Notably, 95 of the 101 patients with auto-Abs were men (94%).

**CONCLUSION:**

A B cell autoimmune phenocopy of inborn errors of type I IFN immunity accounts for life-threatening COVID-19 pneumonia in at least 2.6% of women and 12.5% of men. In these patients, adaptive autoimmunity impairs innate and intrinsic antiviral immunity. These findings provide a first explanation for the excess of men among patients with life-threatening COVID-19 and the increase in risk with age. They also provide a means of identifying individuals at risk of developing life-threatening COVID-19 and ensuring their enrolment in vaccine trials. Finally, they pave the way for prevention and treatment, including plasmapheresis, plasmablast depletion, and recombinant type I IFNs not targeted by the auto-Abs (e.g., IFN-β).

## Teaser

In a large immunological and genomics study of COVID-19 patients, autoantibodies to type 1 interferons correlated with outcomes.

## Graphical abstract

**Figure:**
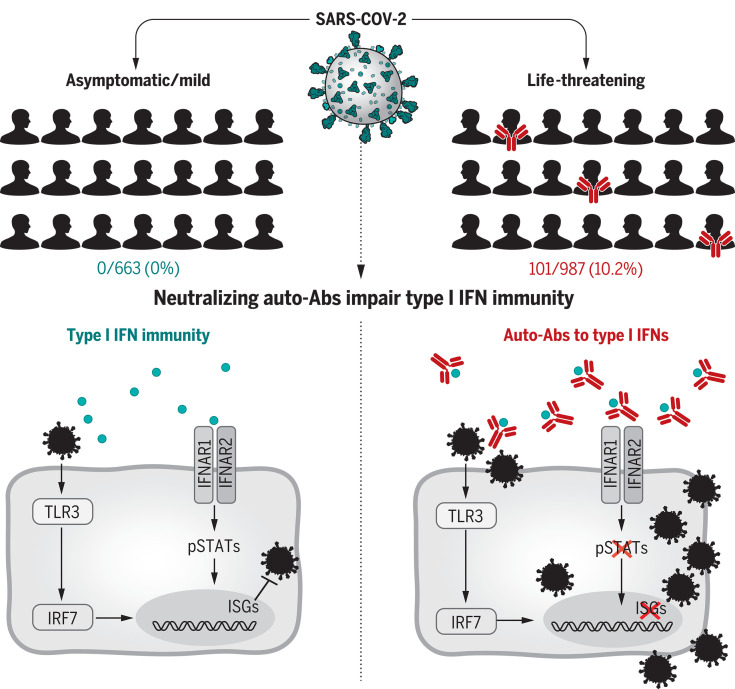
Neutralizing auto-Abs to type I IFNs underlie life-threatening COVID-19 pneumonia. We tested the hypothesis that neutralizing auto-Abs against type I IFNs may underlie critical COVID-19 by impairing the binding of type I IFNs to their receptor and the activation of the downstream responsive pathway. Neutralizing auto-Abs are represented in red, and type I IFNs are represented in blue. In these patients, adaptive autoimmunity impairs innate and intrinsic antiviral immunity. ISGs, IFN-stimulated genes; TLR, Toll-like receptor; IFNAR, IFN-α/β receptor; pSTAT, phosphorylated signal transducers and activators of transcription; IRF, interferon regulatory factor.

Mycobacteriosis, staphylococcosis, and candidiasis can be driven by monogenic inborn errors of interferon-γ (IFN-γ), interleukin-6 (IL-6), and IL-17A and IL-17F, respectively, or they can be driven by their genetically driven autoimmune phenocopies, with the production of neutralizing autoantibodies (auto-Abs) against these cytokines (*1*–*8*). Type I IFNs, first described in 1957, are ubiquitously expressed cytokines that contribute to both innate immunity (through their secretion by plasmacytoid dendritic cells and other leukocytes) and cell-intrinsic immunity (in most if not all cell types) against viral infections (*9*–*13*). Their receptors are ubiquitously expressed and trigger the induction of IFN-stimulated genes (ISGs) via phosphorylated STAT1-STAT2-IRF9 trimers (STAT, signal transducers and activators of transcription; IRF, interferon regulatory factor) (*14*). Neutralizing immunoglobulin G (IgG) auto-Abs against type I IFNs can occur in patients treated with IFN-α2 or IFN-β (*15*) and exist in almost all patients with autoimmune polyendocrinopathy syndrome type I (APS-1) (*16*). They are also seen in women with systemic lupus erythematosus (*17*).

These patients do not seem to suffer from unusually severe viral infections, although human inborn errors of type I IFNs can underlie severe viral diseases, both respiratory and otherwise (*18*). In 1984, Ion Gresser described a patient with unexplained auto-Abs against type I IFNs suffering from severe chickenpox and shingles (*19*, *20*). More recently, auto-Abs against type I IFNs have been found in a few patients with biallelic, hypomorphic *RAG1* or *RAG2* mutations and viral diseases including severe chickenpox and viral pneumonias (*21*). Our attention was drawn to three patients with APS-1, with known preexisting anti–type I IFN auto-Abs, who had life-threatening coronavirus disease 2019 (COVID-19) pneumonia (*22*) (see detailed case reports in Methods). While searching for inborn errors of type I IFNs (*18*, *23*), we hypothesized that neutralizing auto-Abs against type I IFNs might also underlie life-threatening COVID-19 pneumonia.

## Auto-Abs against IFN-α2 and/or IFN-ω in patients with critical COVID-19

**Fig. 1:**
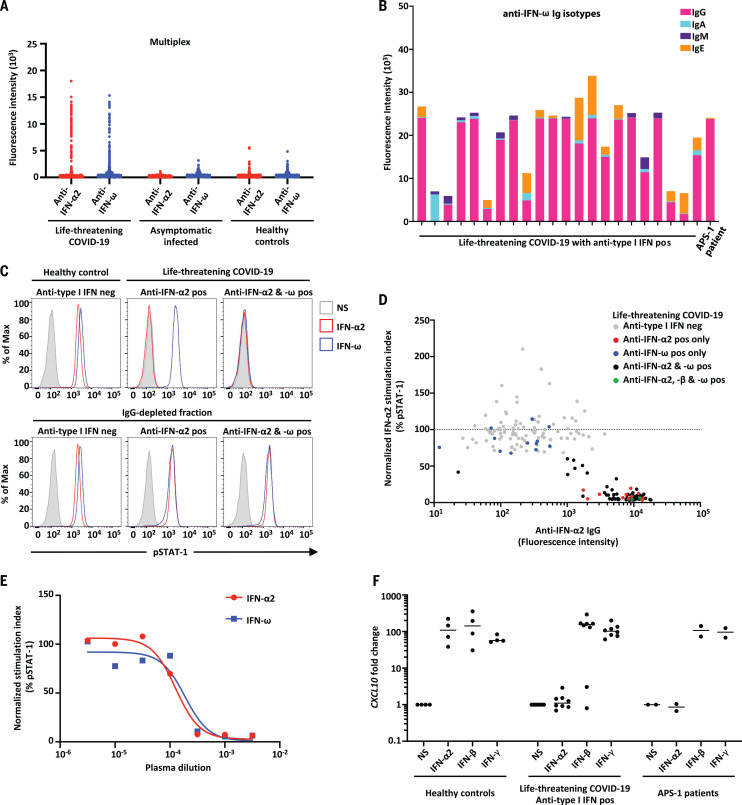
Neutralizing auto-Abs against IFN-α2 and/or IFN-ω in patients with life-threatening COVID-19. (**A**) Multiplex particle-based assay for auto-Abs against IFN-α2 and IFN-ω in patients with life-threatening COVID-19 (*N* = 782), in patients with asymptomatic or mild SARS-CoV-2 infection (*N* = 443), and in healthy controls not infected with SARS-CoV-2 (*N* = 1160). (**B**) Anti–IFN-ω Ig isotypes in 23 patients with life-threatening COVID-19 and auto-Abs to type I IFNs. (**C**) Representative fluorescence-activated cell sorting (FACS) plots depicting IFN-α2– or IFN-ω–induced pSTAT1 in healthy control cells (gated on CD14^+^ monocytes) in the presence of 10% healthy control or anti–IFN-α2 or anti–IFN-ω auto-Abs–containing patient plasma (top panel) or an IgG-depleted plasma fraction (bottom panel). Max, maximum; neg, negative; pos, positive; NS, not stimulated. (**D**) Plot of anti–IFN-α2 auto-Ab levels against their neutralization capacity. The stimulation index (stimulated over unstimulated condition) for the plasma from each patient was normalized against that of healthy control plasma from the same experiment. Spearman’s rank correlation coefficient = −0.6805; *P* < 0.0001. (**E**) Median inhibitory concentration (IC_50_) curves representing IFN-α2– and IFN-ω–induced pSTAT1 levels in healthy donor cells in the presence of serial dilutions of patient plasma. The stimulation index (stimulated over unstimulated condition) for patient plasma was normalized against that of 10% healthy control plasma. IFN-α2: IC_50_ = 0.016%, *R*^2^ = 0.985; IFN-ω: IC_50_ = 0.0353%, *R*^2^ = 0.926. *R*^2^, coefficient of determination. (**F**) Neutralizing effect on *CXLC10* induction, after stimulation with IFN-α2, IFN-β, or IFN-γ, in the presence of plasma from healthy controls (*N* = 4), patients with life-threatening COVID-19 and auto-Abs against IFN-α2 (*N* = 8), and APS-1 patients (*N* = 2).

We searched for auto-Abs against type I IFNs in 987 patients hospitalized for life-threatening COVID-19 pneumonia. We also examined 663 individuals infected with severe acute respiratory syndrome coronavirus 2 (SARS-CoV-2) presenting asymptomatic infection or mild disease and 1227 healthy controls whose samples were collected before the COVID-19 pandemic. Plasma or serum samples were collected from patients with critical COVID-19 during the acute phase of disease. Multiplex particle-based flow cytometry revealed a high fluorescence intensity (FI) (>1500) for IgG auto-Abs against IFN-α2 and/or IFN-ω in 135 patients (13.7%) with life-threatening COVID-19 (Fig. 1A). We found that 49 of these 135 patients were positive for auto-Abs against both IFN-α2 and IFN-ω, whereas 45 were positive only for auto-Abs against IFN-α2, and 41 were positive only for auto-Abs against IFN-ω.

We also performed enzyme-linked immunosorbent assay (ELISA), and the results obtained were consistent with those obtained with Luminex technology (fig. S1A). We found that 11 and 14 of 23 patients tested had low levels of IgM and IgA auto-Abs against IFN-ω and IFN-α2, respectively (Fig. 1B and fig. S1B). Auto-Abs against type I IFNs were detected in two unrelated patients for whom we had plasma samples obtained before SARS-CoV-2 infection, which indicates that these antibodies were present before SARS-CoV-2 infection and were not triggered by the infection. As a control, we confirmed that all 25 APS-1 patients tested had high levels of auto-Abs against IFN-α2 and IFN-ω (fig. S1C). Overall, we found that 135 of 987 patients (13.7%) with life-threatening COVID-19 pneumonia had IgG auto-Abs against at least one type I IFN.

## The auto-Abs neutralize IFN-α2 and IFN-ω in vitro

We then tested whether auto-Abs against IFN-α2 and IFN-ω were neutralizing in vitro. We incubated peripheral blood mononuclear cells (PBMCs) from healthy controls with 10 ng/mL IFN-α2 or IFN-ω in the presence of plasma from healthy individuals or from patients with auto-Abs. A complete abolition of STAT1 phosphorylation was observed in 101 patients with auto-Abs against IFN-α2 and/or IFN-ω (table S1). The antibodies detected were neutralizing against both IFN-α2 and IFN-ω in 52 of these 101 patients (51%), against only IFN-α2 in 36 patients (36%), and against only IFN-ω in 13 patients (13%) at the IFN-α2 and IFN-ω concentrations tested (Fig. 1, C and D). IgG depletion from patients with auto-Abs restored normal pSTAT1 induction after IFN-α2 and IFN-ω stimulation, whereas the purified IgG fully neutralized this induction (Fig. 1C and fig. S1D). Furthermore, these auto-Abs neutralized high amounts of IFN-α2 (fig. S1E) and were neutralizing at high dilutions (Fig. 1E and fig. S1F). Notably, 15 patients with life-threatening COVID-19 and auto-Abs against IFN-α2 and/or IFN-ω also had auto-Abs against other cytokines [IFN-γ, granulocyte-macrophage colony-stimulating factor (GM-CSF), IL-6, IL-10, IL-12p70, IL-22, IL-17A, IL-17F, and/or tumor necrosis factor–β (TNFβ)], only three of which (IL-12p70, IL-22, and IL-6) were neutralizing (in four patients) (fig. S2, A to C). Similar proportions were observed in the other cohorts (fig. S2, D to L).

We also analyzed ISG induction after 2 hours of stimulation with IFN-α2, IFN-β, or IFN-γ in the presence of plasma from healthy individuals or from patients with auto-Abs. With plasma from eight patients with auto-Abs against IFN-α2, the induction of ISG *CXCL10* was abolished after IFN-α2 stimulation but maintained after stimulation with IFN-γ (Fig. 1F). We then found that plasma from the five patients with neutralizing auto-Abs neutralized the protective activity of IFN-α2 in Madin–Darby bovine kidney (MDBK) cells infected with vesicular stomatitis virus (VSV) (table S2). Overall, we found that 101 of 987 patients (10.2%)—including 95 men (94%)—with life-threatening COVID-19 pneumonia had neutralizing IgG auto-Abs against at least one type I IFN. By contrast, auto-Abs were detected in only 4 of 1227 healthy controls (0.33%) (Fisher exact test, *P* < 10^−16^) and in none of the 663 patients with asymptomatic or mild SARS-CoV-2 infection tested (Fisher exact test, *P* < 10^−16^).

## Auto-Abs against all 13 IFN-α subtypes in patients with auto-Abs to IFN-α2

**Fig. 2:**
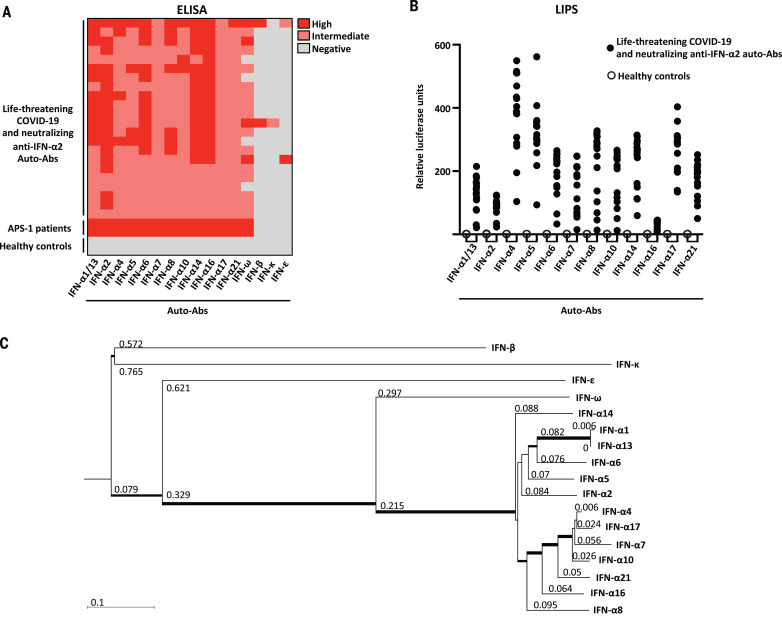
Auto-Abs against the different type I IFN subtypes. (**A**) ELISA for auto-Abs against the 13 different IFN-α subtypes, IFN-ω, IFN-β, IFN-κ, and IFN-ε in patients with life-threatening COVID-19 and auto-Abs against IFN-α2 (*N* = 22), APS-1 patients (*N* = 2), and healthy controls (*N* = 2). (**B**) LIPS for the 12 different IFN-α subtypes tested in patients with auto-Abs against IFN-α2 (*N* = 22) and healthy controls (*N* = 2). (**C**) Neighbor-joining phylogenetic tree of the 17 human type I IFN proteins. Horizontal branches are drawn to scale (bottom left, number of substitutions per site). Thinner, intermediate, and thicker internal branches have bootstrap support of <50, ≥50, and >80%, respectively. The bootstrap value for the branch separating IFN-ω from all IFN-α subtypes is 100%.

We investigated whether patients with neutralizing auto-Abs against IFN-α2 only or those with neutralizing auto-Abs against IFN-α2 and IFN-ω also had auto-Abs against the other 15 type I IFNs. ELISA showed that all patients tested (*N* = 22) with auto-Abs against IFN-α2 also had auto-Abs against all 13 IFN-α subtypes (IFN-α1, -α2, -α4, -α5, -α6, -α7, -α8, -α10, -α13, -α14, -α16, -α17, and -α21), whereas only 2 of the 22 patients tested had auto-Abs against IFN-β, 1 had auto-Abs against IFN-κ, and 2 had auto-Abs against IFN-ε (Fig. 2A). The auto-Abs against IFN-β had neutralizing activity against IFN-β (Fig. 1D). We confirmed that all of the patients had auto-Abs against all 13 subtypes of IFN-α by testing the same samples using luciferase-based immunoprecipitation assay (LIPS) (Fig. 2B). For IFN-β, we also screened the whole cohort in a multiplex assay. We found that 19 of 987 (1.9%) patients had auto-Abs against IFN-β and that all of them were in our cohort of severe COVID-19 individuals with neutralizing auto-Abs against IFN-α and/or IFN-ω. Of these patients with auto-Abs against IFN-β, only two were neutralizing against IFN-β (Fig. 1, D and F).

Ten of the 17 genes encoding type I IFNs (IFN-α2, -α5, -α6, α8, -α13, -α14, -α21, -β, -ω, and -κ), have undergone strong negative selection, which suggests that they play an essential role in the general population. By contrast, the other seven IFN loci in the human genome often carry loss-of-function alleles (*24*). Moreover, the 13 IFN-α subtypes and IFN-ω are more-closely related to each other than they are to the other three IFNs (IFN-β, IFN-ε, and IFN-κ), which are structurally and phylogenetically more distant (Fig. 2C). Thus, all patients with neutralizing auto-Abs against IFN-α2 that we tested (*N* = 22) had auto-Abs against all 13 IFN-α subtypes, and 3 of the 22 patients tested (14%) had auto-Abs against 14 or more type I IFNs.

## The auto-Abs neutralize IFN-α2 against SARS-CoV-2 in vitro and IFN-α in vivo

**Fig. 3:**
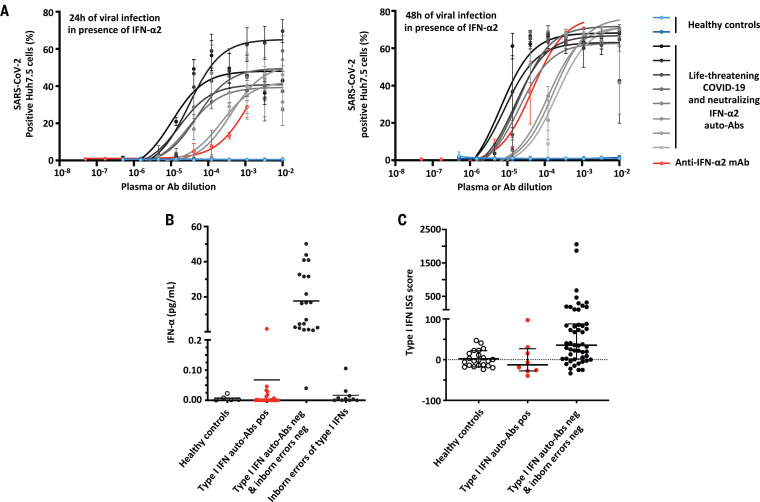
Enhanced SARS-CoV-2 replication, despite the presence of IFN-α2, in the presence of plasma from patients with auto-Abs against IFN-α2 and low in vivo levels of IFN-α. (**A**) SARS-CoV-2 replication—measured 24 hours (left) and 48 hours (right) after infection—in Huh7.5 cells treated with IFN-α2 in the presence of plasma from patients with life-threatening COVID-19 and neutralizing auto-Abs against IFN-α2 (*N* = 8); a commercial anti–IFN-α2 antibody; or control plasma (*N* = 2). (**B**) IFN-α levels in the plasma or serum of patients with neutralizing auto-Abs (*N* = 41), healthy controls (*N* = 5), COVID-19 patients without auto-Abs (*N* = 21), and patients with life-threatening COVID-19 and loss-of-function (LOF) variants (*N* = 10), as assessed by Simoa ELISA. (**C**) *z*-scores for type I IFN gene responses in whole blood of COVID-19 patients with (*N* = 8) or without (*N* = 51) neutralizing auto-Abs, or healthy uninfected controls (*N* = 22). The median ± interquartile range is shown. *z*-scores were significantly lower for patients with neutralizing auto-Abs compared with patients without auto-Abs (Mann-Whitney test, *P* = 0.01).

Plasma from eight patients with neutralizing auto-Abs against type I IFN also neutralized the ability of IFN-α2 to block the infection of Huh7.5 cells with SARS-CoV-2 (Fig. 3A). Plasma from two healthy controls or from seven SARS-CoV-2–infected patients without auto-Abs did not block the protective action of IFN-α2 (Fig. 3A and fig. S3A). These data provide compelling evidence that the patients’ blood carried sufficiently large amounts of auto-Abs to neutralize the corresponding type I IFNs and block their antiviral activity in vitro, including that against SARS-CoV-2.

We also found that all 41 patients with neutralizing auto-Abs against the 13 types of IFN-α tested had low (one patient) or undetectable (40 patients) levels of the 13 types of IFN-α in their plasma during the course of the disease (Fig. 3B) (*25*, *26*). Type I IFNs may be degraded and/or bound to the corresponding circulating auto-Abs. The presence of circulating neutralizing auto-Abs against IFN-α is, therefore, strongly associated with low serum IFN-α levels (Fisher exact test, *P* < 10^−6^). Consistently in patients with neutralizing auto-Abs against IFN-α2, the baseline levels of type I IFN–dependent transcripts were low, whereas they were normal for nuclear factor κB (NF-κB)–dependent transcripts (Fig. 3C and fig. S3B). Overall, our findings indicate that the auto-Abs against type I IFNs present in patients with life-threatening COVID-19 were neutralizing in vitro and in vivo.

## Pronounced excess of men in patients with auto-Abs against type I IFNs

**Fig. 4:**
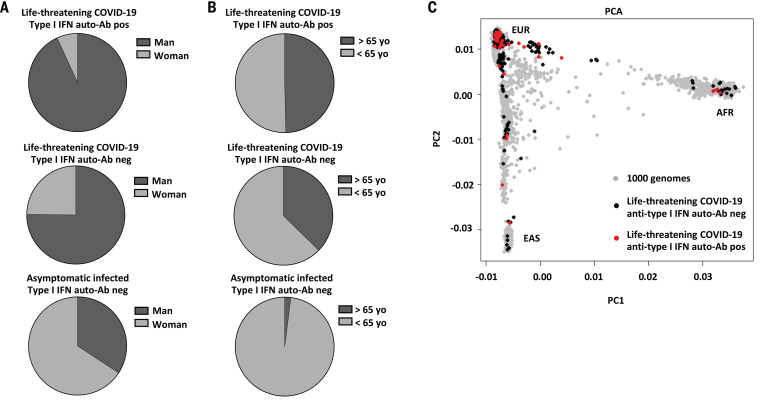
Demographic and ethnic information about the patients and controls. (**A**) Gender distribution in patients with life-threatening COVID-19 and auto-Abs to type I IFNs, patients with life-threatening COVID-19 and without auto-Abs to type I IFNs, and individuals with asymptomatic or mild SARS-CoV-2. (**B**) Age distribution in patients with life-threatening COVID-19 and auto-Abs to type I IFNs, patients with life-threatening COVID-19 and without auto-Abs to type I IFNs, and individuals with asymptomatic or mild SARS-CoV-2. yo, years old. (**C**) PCA on 49 patients with life-threatening COVID-19 and auto-Abs against type I IFNs. EUR, Europeans; AFR, Africans; EAS, East-Asians.

There was a pronounced excess of male patients (95 of 101; 94%) with critical COVID-19 pneumonia and neutralizing auto-Abs against type I IFNs. This proportion of males was higher than that observed in patients with critical COVID-19 without auto-Abs (75%; Fisher exact test, *P* = 2.5 × 10^−6^), and the proportion was much higher than that in male patients in the asymptomatic or pauci-symptomatic cohort (28%; Fisher exact test, *P* < 10^−6^) (Table 1, Fig. 4A, and fig. S4A). Further evidence for X-chromosome linkage was provided by the observation that one of the seven women with auto-Abs and life-threatening COVID-19 had X chromosome–linked incontinentia pigmenti (IP), in which cells activate only a single X chromosome (cells having activated the X chromosome bearing the null mutation in *NEMO* dying in the course of development) (*27*). The prevalence of auto-Abs against type I IFNs in the general population was estimated at 0.33% (0.015 to 0.67%) in a sample of 1227 healthy individuals—a value much lower than that in patients with life-threatening COVID-19 pneumonia, by a factor of at least 15.

**Table 1:** Sex and age distribution of patients with critical COVID-19 with and without auto-Abs. Ages and sexes of the patients and controls and information about auto-Abs against IFN-α2 and IFN-ω, presented by age and sex. Dashes in rightmost column indicate data not available. OR, odds ratio; CI, confidence interval.

**Life-threatening** **COVID-19**	***N* total**	***N* auto-Abs positive** **(percentage)**	**OR [95% CI]**	*P* value*
*Sex*				
Female	226	6 (2.6%)	1	–
Male	761	95 (12.5%)	5.22 [2.27 – 14.80]	2.5 × 10^−6^
*Age*				
<65 years	602	51 (8.5%)	1	–
≥65 years	385	50 (13.0%)	1.61 [1.04 – 2.49]	0.024

*P values were derived from Fisher’s exact test, as implemented in R (https://cran.r-project.org/).

The patients with auto-Abs were also slightly older than the rest of our cohort (49.5% of patients positive for auto-Abs were over 65 years of age versus 38% for the rest of the cohort; *P* = 0.024), which suggests that the frequency of circulating anti–type I IFNs auto-Abs increases with age (Table 1 and Fig. 4B). However, auto-Abs were found in patients aged from 25 to 87 years (fig. S4B). Principal components analysis (PCA) was performed on data from 49 patients: 34 Europeans, 5 North Africans, 4 sub-Saharan Africans, 2 patients from the Middle East, 2 South Asians, 1 East Asian, and 1 South American (Fig. 4C). Large-scale studies will be required to determine the frequency of such auto-Abs in humans of different sexes, ages, and ancestries. Finally, the presence of auto-Abs was associated with a poor outcome, with death occurring in 37 of the 101 patients (36.6%) (table S1).

## Neutralizing auto-Abs to type I IFNs are causative of critical COVID-19

There are multiple lines of evidence to suggest that the neutralizing auto-Abs against type I IFNs observed in these 101 patients preceded infection with SARS-CoV-2 and accounted for the severity of disease. First, the two patients for whom testing was performed before COVID-19 were found to have auto-Abs before infection. Second, three patients with APS-1 known to have neutralizing auto-Abs against type I IFN immunity before infection also had life-threatening COVID-19 (*22*) (supplementary methods). Third, we screened a series of 32 women with IP and found that a quarter of them had auto-Abs against type I IFNs, including one who developed critical COVID-19 (fig. S1C). Fourth, there is a marked bias in favor of men, which suggests that the production of auto-Abs against type I IFNs—whether driven by germ line or somatic genome—may be X chromosome–linked and therefore preexisting to infection.

Moreover, IFN-α subtypes were undetectable during acute disease in the blood of patients with auto-Abs against IFN-α, which suggests a preexisting or concomitant biological impact in vivo. It is also unlikely that patients could break self-tolerance and mount high titers of neutralizing IgG auto-Abs against type I IFN within only 1 or even 2 weeks of infection. Finally, inborn errors of type I IFNs underlying life-threatening COVID-19 in other previously healthy adults—including autosomal recessive IFN-α/β receptor subunit 1 (IFNAR1) deficiency—have also been reported in an accompanying paper (*18*). Collectively, these findings suggest that auto-Abs against type I IFNs are a cause and not a consequence of severe SARS-Cov-2 infection, although their titers and affinity may be enhanced by the SARS-CoV-2–driven induction of type I IFNs. They also provide an explanation for the major sex bias seen in patients with life-threatening COVID-19 and perhaps also for the increase in risk with age.

## Conclusion

We report here that at least 10% of patients with life-threatening COVID-19 pneumonia have neutralizing auto-Abs against type I IFNs. With our accompanying description of patients with inborn errors of type I IFNs and life-threatening COVID-19 (*18*), this study highlights the crucial role of type I IFNs in protective immunity against SARS-CoV-2. These auto-Abs against type I IFNs were clinically silent until the patients were infected with SARS-CoV-2—a poor inducer of type I IFNs (*28*)—which suggests that the small amounts of IFNs induced by the virus are important for protection against severe disease. The neutralizing auto-Abs against type I IFNs, like inborn errors of type I IFN production, tip the balance in favor of the virus, which results in devastating disease with insufficient, and even perhaps deleterious, innate and adaptive immune responses.

Our findings have direct clinical implications. First, SARS-CoV-2–infected patients can be screened to identify individuals with auto-Abs at risk of developing life-threatening pneumonia. Such patients recovering from life-threatening COVID-19 should also be excluded from donating convalescent plasma for ongoing clinical trials, or at least they should be tested before their plasma donations are accepted (*29*). Second, this finding paves the way for preventive or therapeutic intervention, including plasmapheresis, monoclonal Abs depleting plasmablasts, and the specific inhibition of type I IFN–reactive B cells (*30*). Finally, in this patient group, early treatment with IFN-α is unlikely to be beneficial; however, treatment with injected or nebulized IFN-β may have beneficial effects, as auto-Abs against IFN-β appear to be rare in patients with auto-Abs against type I IFNs.

## Materials and methods

### Subjects and samples

We enrolled 987 patients with proven life-threatening (critical) COVID-19, 663 asymptomatic or pauci-symptomatic individuals with proven COVID-19, and 1227 healthy controls in this study. All subjects were recruited following protocols approved by local Institutional Review Boards (IRBs). All protocols followed local ethics recommendations and informed consent was obtained when required.

COVID-19 disease severity was assessed in accordance with the Diagnosis and Treatment Protocol for Novel Coronavirus Pneumonia. The term life-threatening COVID-19 pneumonia describes pneumonia in patients with critical disease, whether pulmonary, with mechanical ventilation [continuous positive airway pressure (CPAP), bilevel positive airway pressure (BIPAP), intubation, or high-flow oxygen], septic shock, or damage to any other organ requiring admission in the intensive care unit (ICU). The individuals with asymptomatic or mild SARS-CoV-2 infection were individuals infected with SARS-CoV-2 who remained asymptomatic or developed mild, self-healing, ambulatory disease with no evidence of pneumonia. The healthy controls were individuals who had not been exposed to SARS-CoV-2.

Plasma and serum samples from the patients and controls were frozen at −20°C immediately after collection. The fluid-phase LIPS assay was used to determine the levels of antibodies against the SARS-CoV-2 nucleoprotein and spike protein, as has been previously described (*31*).

### Detection of anti-cytokine auto-Abs

#### Multiplex particle-based assay

Serum and plasma samples were screened for auto-Abs against 18 targets in a multiplex particle-based assay, in which magnetic beads with differential fluorescence were covalently coupled to recombinant human proteins. Patients with an FI of >1500 for IFN-α2 or IFN-β or >1000 for IFN-ω were tested for blocking activity, as were patients positive for another cytokine.

#### ELISA

ELISA was performed as previously described (*5*). In brief, ELISA plates were coated with recombinant human interferon-α (rhIFN-α) or rhIFN-ω and incubated with 1:50 dilutions of plasma samples from the patients or controls. A similar protocol was used when testing for 12 subtypes of IFN-α.

#### LIPS

Levels of auto-Abs against IFN-α subtypes were measured with LIPS, as previously described (*32*). IFN-α1, IFN-α2, IFN-α4, IFN-α5, IFN-α6, IFN-α7, IFN-α8, IFN-α10, IFN-α14, IFN-α16, IFN-α17, and IFN-α21 sequences were transfected in HEK293 cells, and the IFN-α-luciferase fusion proteins were collected in the tissue culture supernatant. For autoantibody screening, serum samples were incubated with protein G agarose beads, and we then added 2 × 10^6^ luminescence units (LU) of antigen and incubated. Luminescence intensity was measured. The results are expressed in arbitrary units (AU), as a fold-difference relative to the mean of the negative control samples.

### Functional evaluation of anti-cytokine auto-Abs

The blocking activity of anti–IFN-α and anti–IFN-ω auto-Abs was determined by assessing STAT1 phosphorylation in healthy control cells after stimulation with the appropriate cytokines in the presence of 10% healthy control or patient serum or plasma.

We demonstrated that the IFN-α and IFN-ω blocking activity observed was due to auto-Abs and not another plasma factor, by depleting IgG from the plasma with a protein G column Without eluting the IgG, the flow-through fraction (IgG-depleted) was then collected and compared with total plasma in the phospho-STAT1 assay.

The blocking activity of anti–IFN-γ, –GM-CSF, –IFN-λ1, –IFN-λ2, –IFN-λ3, –IL-6, –IL-10, –IL-12p70, –IL-22, –IL-17A, –IL-17F, -TNFα, and -TNFβ antibodies was assessed with the assays outlined in table S3, as previously reported (*21*).

For the neutralization of ISG induction, PBMCs were left unstimulated or were stimulated for 2 hours with 10 ng/mL IFN-α or 10 ng/mL IFN-γ in a final volume of 100 μL. Real-time quantitative polymerase chain reaction (RT-qPCR) analysis was performed with Applied Biosystems *Taq*man assays for *CXCL10*, and the β-glucuronidase (GUS) housekeeping gene for normalization. Results are expressed according to the ΔΔCt method, as described by the manufacturer’s kit.

### Phylogenetic reconstruction

Protein sequences were aligned with the online version of MAFFT v7.471 software (*33*), using the L-INS-i strategy (*34*) and the BLOSUM62 scoring matrix for amino acid substitutions. Phylogenetic tree reconstruction was performed by the neighbor-joining method (*35*) with the substitution model (*36*). Low-confidence branches (<50%) are likely to be due to gene conversion events between *IFNA* genes, as previously reported (*24*, *37*). The tree was then visualized (*38*). Very similar results were obtained with the corresponding DNA sequences (*37*, *39*).

### Statistical analysis

Comparison of proportions were performed using a Fisher exact test, as implemented in R (https://cran.r-project.org/). PCA was performed with Plink v1.9 software on whole-exome and whole-genome sequencing data with the 1000 Genomes (1kG) Project phase 3 public database as a reference.

### Simoa

Serum IFN-α concentrations were determined with Simoa technology, as previously described (*40*, *41*), with reagents and procedures obtained from the Quanterix Corporation.

### VSV assay

The seroneutralization assay was performed as previously described (*42*). In brief, the incubation of IFN-α2 with MDBK cells protects the cultured cells against the cytopathic effect of VSV. The titer of anti–IFN-α antibodies was defined as the last dilution causing 50% cell death.

### SARS-CoV-2 experiment

SARS-CoV-2 strain USA-WA1/2020 was obtained from BEI Resources and amplified in Huh7.5 hepatoma cells at 33°C. Viral titers were measured on Huh7.5 cells in a standard plaque assay. Plasma samples or a commercial anti–IFN-α2 antibody were serially diluted and incubated with 20 pM recombinant IFN-α2 for 1 hour at 37°C (starting concentrations: plasma samples = 1/100 and anti–IFN-α2 antibody = 1/1000). The cell culture medium was then removed and replaced with the plasma– or antibody–IFN-α2 mixture. The plates were incubated overnight, and the plasma– or antibody–IFN-α2 mixture was removed by aspiration. The cells were washed once with phosphate-buffered saline (PBS) to remove potential anti–SARS-CoV-2 neutralizing antibodies, and fresh medium was then added. Cells were then infected with SARS-CoV-2 by directly adding the virus to the wells. Cells infected at a high multiplicity of infection (MOI) were incubated at 37°C for 24 hours, whereas cells infected at a low MOI were incubated at 33°C for 48 hours. The cells were fixed with 7% formaldehyde, stained for SARS-CoV-2 with an anti-N antibody, imaged, and analyzed as previously described (*43*).

### Nanostring

For the NanoString assay, total RNA was extracted from whole blood samples collected in PaxGene tubes. The expression of selected genes was determined by NanoString methods and a 28-gene type I IFN score was calculated (*44*).

## Supplementary Materials

science.sciencemag.org/content/370/6515/eabd4585/suppl/DC1 Supplementary Materials and Methods Figs. S1 to S4 Tables S1 to S3 Data S1

## Appendix

View/request a protocol for this paper from *Bio-protocol*.

## HGID Lab

Andrés Augusto Arias^1,3^, Bertrand Boisson^1,2^, Soraya Boucherit^2^, Jacinta Bustamante^1,2^, Marwa Chbihi^2^, Jie Chen^1^, Maya Chrabieh^2^, Tatiana Kochetkov^1^, Tom Le Voyer^2^, Dana Liu^1^, Yelena Nemirovskaya^1^, Masato Ogishi^1^, Dominick Papandrea^1^, Cécile Patissier^2^, Franck Rapaport^1^, Manon Roynard^2^, Natasha Vladikine^2^, Mark Woollett^1^, Peng Zhang^1^

^1^St. Giles Laboratory of Human Genetics of Infectious Diseases, Rockefeller Branch, The Rockefeller University, New York, NY, USA. ^2^Laboratory of Human Genetics of Infectious Diseases, Necker Branch, INSERM U1163, Necker Hospital for Sick Children, Paris, France. ^3^School of Microbiology and Group of Primary Immunodeficiencies, University of Antioquia UdeA, Medellín, Colombia.

## NIAID-USUHS Immune Response to COVID Group

Anuj Kashyap^1^, Li Ding^1^, Marita Bosticardo^1^, Qinlu Wang^2^, Sebastian Ochoa^1^, Hui Liu^1^, Samuel D. Chauvin^3^, Michael Stack^1^, Galina Koroleva^4^, Neha Bansal^5^, Clifton L. Dalgard^6,7^, Andrew L. Snow^8^

^1^Laboratory of Clinical Immunology and Microbiology, Division of Intramural Research, NIAID, NIH, Bethesda, MD, USA. ^2^Bioinformatics and Computational Biosciences Branch, NIAID Office of Cyber Infrastructure and Computational Biology, NIAID, NIH, Bethesda, MD, USA. ^3^Laboratory of Immune System Biology, Division of Intramural Research, NIAID, NIH, Bethesda, MD, USA. ^4^NIH Center for Human Immunology, NIH, Bethesda, MD, USA. ^5^Multiscale Systems Biology Section, Laboratory of Immune System Biology, NIAID, NIH, Bethesda, MD, USA. ^6^PRIMER, Uniformed Services University of the Health Sciences, Bethesda, MD, USA. ^7^Department of Anatomy, Physiology & Genetics, Uniformed Services University of the Health Sciences, Bethesda, MD, USA. ^8^Department of Pharmacology & Molecular Therapeutics, Uniformed Services University of the Health Sciences, Bethesda, MD, USA.

## COVID Clinicians

Jorge Abad^1^, Sergio Aguilera-Albesa^2^, Ozge Metin Akcan^3^, Ilad Alavi Darazam^4^, Juan C. Aldave^5^, Miquel Alfonso Ramos^6^, Seyed Alireza Nadji^7^, Gulsum Alkan^8^, Jerome Allardet-Servent^9^, Luis M. Allende^10^, Laia Alsina^11^, Marie-Alexandra Alyanakian^12^, Blanca Amador-Borrero^13^, Zahir Amoura^14^, Arnau Antolí^15^, Sevket Arslan^16^, Sophie Assant^17^, Terese Auguet^18^, Axelle Azot^19^, Fanny Bajolle^20^, Aurélie Baldolli^21^, Maite Ballester^22^, Hagit Baris Feldman^23^, Benoit Barrou^24^, Alexandra Beurton^25^, Agurtzane Bilbao^26^, Geraldine Blanchard-Rohner^27^, Ignacio Blanco^1^, Adeline Blandinières^28^, Daniel Blazquez-Gamero^29^, Marketa Bloomfield^30^, Mireia Bolivar-Prados^31^, Raphael Borie^32^, Ahmed A. Bousfiha^33^, Claire Bouvattier^34^, Oksana Boyarchuk^35^, Maria Rita P. Bueno^36^, Jacinta Bustamante^20^, Juan José Cáceres Agra^37^, Semra Calimli^38^, Ruggero Capra^39^, Maria Carrabba^40^, Carlos Casasnovas^41^, Marion Caseris^42^, Martin Castelle^43^, Francesco Castelli^44^, Martín Castillo de Vera^45^, Mateus V. Castro^36^, Emilie Catherinot^46^, Martin Chalumeau^47^, Bruno Charbit^48^, Matthew P. Cheng^49^, Père Clavé^31^, Bonaventura Clotet^50^, Anna Codina^51^, Fatih Colkesen^52^, Fatma Colkesen^53^, Roger Colobran ^54^, Cloé Comarmond^55^, Angelo G. Corsico^56^, David Dalmau^57^, David Ross Darley^58^, Nicolas Dauby^59^, Stéphane Dauger^60^, Loic de Pontual^61^, Amin Dehban^62^, Geoffroy Delplancq^63^, Alexandre Demoule^64^, Antonio Di Sabatino^65^, Jean-Luc Diehl^66^, Stephanie Dobbelaere^67^, Sophie Durand^68^, Waleed Eldars^69^, Mohamed Elgamal^70^, Marwa H. Elnagdy^71^, Melike Emiroglu^72^, Emine Hafize Erdeniz^73^, Selma Erol Aytekin^74^, Romain Euvrard^75^, Recep Evcen^76^, Giovanna Fabio^40^, Laurence Faivre^77^, Antonin Falck^42^, Muriel Fartoukh^78^, Morgane Faure^79^, Miguel Fernandez Arquero^80^, Carlos Flores^81^, Bruno Francois^82^, Victoria Fumadó^83^, Francesca Fusco^84^, Blanca Garcia Solis^85^, Pascale Gaussem^86^, Juana Gil-Herrera^87^, Laurent Gilardin^88^, Monica Girona Alarcon^89^, Mónica Girona-Alarcón^89^, Jean-Christophe Goffard^90^, Funda Gok^91^, Rafaela González-Montelongo^92^, Antoine Guerder^93^, Yahya Gul^94^, Sukru Nail Guner^94^, Marta Gut^95^, Jérôme Hadjadj^96^, Filomeen Haerynck^97^, Rabih Halwani^98^, Lennart Hammarström^99^, Nevin Hatipoglu^100^, Elisa Hernandez-Brito^101^, María Soledad Holanda-Peña^102^, Juan Pablo Horcajada^103^, Sami Hraiech^104^, Linda Humbert^105^, Alejandro D. Iglesias^106^, Antonio Íñigo-Campos^92^, Matthieu Jamme^107^, María Jesús Arranz^108^, Iolanda Jordan^109^, Fikret Kanat^110^, Hasan Kapakli^111^, Iskender Kara^112^, Adem Karbuz^113^, Kadriye Kart Yasar^114^, Sevgi Keles^115^, Yasemin Kendir Demirkol^116^, Adam Klocperk^117^, Zbigniew J. Król^118^, Paul Kuentz^119^, Yat Wah M. Kwan^120^, Jean-Christophe Lagier^121^, Yu-Lung Lau^122^, Fleur Le Bourgeois^60^, Yee-Sin Leo^123^, Rafael Leon Lopez^124^, Daniel Leung^122^, Michael Levin^125^, Michael Levy^60^, Romain Lévy^20^, Zhi Li^48^, Agnes Linglart^126^, José M. Lorenzo-Salazar^92^, Céline Louapre^127^, Catherine Lubetzki^127^, Charles-Edouard Luyt^128^, David C. Lye^129^, Davood Mansouri^130^, Majid Marjani^131^, Jesus Marquez Pereira^132^, Andrea Martin^133^, David Martínez Pueyo^134^, Javier Martinez-Picado^135^, Iciar Marzana^136^, Alexis Mathian^14^, Larissa R. B. Matos^36^, Gail V. Matthews^137^, Julien Mayaux^138^, Jean-Louis Mège^139^, Isabelle Melki^140^, Jean-François Meritet^141^, Ozge Metin^142^, Isabelle Meyts^143^, Mehdi Mezidi^144^, Isabelle Migeotte^145^, Maude Millereux^146^, Tristan Mirault^147^, Clotilde Mircher^68^, Mehdi Mirsaeidi^148^, Abián Montesdeoca Melián^149^, Antonio Morales Martinez^150^, Pierre Morange^151^, Clémence Mordacq^105^, Guillaume Morelle^152^, Stéphane Mouly^13^, Adrián Muñoz-Barrera^92^, Cyril Nafati^153^, João Farela Neves^154^, Lisa F. P. Ng^155^, Yeray Novoa Medina^156^, Esmeralda Nuñez Cuadros^157^, J. Gonzalo Ocejo-Vinyals^158^, Zerrin Orbak^159^, Mehdi Oualha^20^, Tayfun Özçelik^160^, Qiang Pan Hammarström^161^, Christophe Parizot^138^, Tiffany Pascreau^162^, Estela Paz-Artal^163^, Rebeca Pérez de Diego^85^, Aurélien Philippe^164^, Quentin Philippot^78^, Laura Planas-Serra^165^, Dominique Ploin^166^, Julien Poissy^167^, Géraldine Poncelet^42^, Marie Pouletty^168^, Paul Quentric^138^, Didier Raoult^139^, Anne-Sophie Rebillat^68^, Ismail Reisli^169^, Pilar Ricart^170^, Jean-Christophe Richard^171^, Nadia Rivet^28^, Jacques G. Rivière^172^, Gemma Rocamora Blanch^15^, Carlos Rodrigo^1^, Carlos Rodriguez-Gallego^173^, Agustí Rodríguez-Palmero^174^, Carolina Soledad Romero^175^, Anya Rothenbuhler^176^, Flore Rozenberg^177^, Maria Yolanda Ruiz del Prado^178^, Joan Sabater Riera^15^, Oliver Sanchez^179^, Silvia Sánchez-Ramón^180^, Agatha Schluter^165^, Matthieu Schmidt^181^, Cyril E. Schweitzer^182^, Francesco Scolari^183^, Anna Sediva^184^, Luis M. Seijo^185^, Damien Sene^13^, Sevtap Senoglu^114^, Mikko R. J. Seppänen^186^, Alex Serra Ilovich^187^, Mohammad Shahrooei^62^, David Smadja^188^, Ali Sobh^189^, Xavier Solanich Moreno^15^, Jordi Solé-Violán^190^, Catherine Soler^191^, Pere Soler-Palacín^133^, Yuri Stepanovskiy^192^, Annabelle Stoclin^193^, Fabio Taccone^145^, Yacine Tandjaoui-Lambiotte^194^, Jean-Luc Taupin^195^, Simon J. Tavernier^196^, Benjamin Terrier^197^, Caroline Thumerelle^105^, Gabriele Tomasoni^198^, Julie Toubiana^47^, Josep Trenado Alvarez^199^, Sophie Trouillet-Assant^200^, Jesús Troya^201^, Alessandra Tucci^202^, Matilde Valeria Ursini‬^84^, Yurdagul Uzunhan^203^, Pierre Vabres^204^, Juan Valencia-Ramos^205^, Ana Maria Van Den Rym^85^, Isabelle Vandernoot^206^, Hulya Vatansev^207^, Valentina Vélez-Santamaria^41^, Sébastien Viel^166^, Cédric Vilain^208^, Marie E. Vilaire^68^, Audrey Vincent^34^, Guillaume Voiriot^209^, Fanny Vuotto^105^, Alper Yosunkaya^91^, Barnaby E. Young^123^, Fatih Yucel^210^, Faiez Zannad^211^, Mayana Zatz^36^, Alexandre Belot^212^*

^1^University Hospital and Research Institute “Germans Trias i Pujol”, Badalona, Spain. ^2^Navarra Health Service Hospital, Pamplona, Spain. ^3^Division of Pediatric Infectious Diseases, Necmettin Erbakan University, Meram Medical Faculty, Konya, Turkey. ^4^Department of Infectious Diseases, Loghman Hakim Hospital, Shahid Beheshti University of Medical Sciences, Tehran, Iran. ^5^Hospital Nacional Edgardo Rebagliati Martins, Lima, Peru. ^6^Parc Sanitari Sant Joan de Déu, Sant Boi de Llobregat, Barcelona, Spain. ^7^Virology Research Center, National institutes of Tuberculosis and Lung diseases, Shahid Beheshti University of Medical Sciences, Tehran, Iran. ^8^Division of Pediatric Infectious Diseases, Faculty of Medicine, Selcuk University, Konya, Turkey. ^9^Intensive Care Unit, Hôpital Européen, Marseille, France. ^10^Immunology Department, University Hospital 12 de Octubre, Research Institute imas12, Complutense University, Madrid, Spain. ^11^Hospital Sant Joan de Déu, Barcelona, Spain. ^12^Department of Biological Immunology, Necker Hospital for Sick Children, APHP and INEM, Paris, France. ^13^Internal Medicine Department, Hôpital Lariboisière, APHP; Université de Paris, Paris, France. ^14^Internal Medicine Department, Pitié-Salpétrière Hospital, Paris, France. ^15^Hospital Universitari de Bellvitge, Barcelona, Spain. ^16^Division of Clinical Immunology and Allergy, Necmettin Erbakan University, Meram Medical Faculty, Konya, Turkey. ^17^Joint Research Unit, Hospices Civils de Lyon-bio Mérieux, Hospices Civils de Lyon, Lyon Sud Hospital, Lyon, France. ^18^Hospital Universitario de Tarragona Joan XXIII, Universitat Rovira i Virgili (URV), IISPV, Tarragona, Spain. ^19^Private practice, Paris, France. ^20^Necker Hospital for Sick Children, AP-HP, Paris, France. ^21^Department of Infectious Diseases, CHU de Caen, Caen, France. ^22^Consorcio Hospital General Universitario, Valencia, Spain. ^23^The Genetics Institute, Tel Aviv Sourasky Medical Center and Sackler Faculty of Medicine, Tel Aviv University, Tel Aviv, Israel. ^24^Department of Urology, Nephrology, Transplantation, APHP-SU, Sorbonne Université, INSERM U 1082, Paris, France. ^25^Service de Médecine Intensive–Réanimation et Pneumologie, APHP Hôpital Pitié–Salpêtrière, Paris, France. ^26^Cruces University Hospital, Bizkaia, Spain. ^27^Paediatric Immunology and Vaccinology Unit, Geneva University Hospitals and Faculty of Medicine, Geneva, Switzerland. ^28^Hematology, Georges Pompidou Hospital, APHP, Paris, France. ^29^Pediatric Infectious Diseases Unit, Instituto de Investigación 12 de Octubre (imas12), Hospital Universitario 12 de Octubre, Madrid, Spain. ^30^Department of Immunology, Motol University Hospital, 2nd Faculty of Medicine, Charles University, Department of Pediatrics, Thomayer’s Hospital, 1st Faculty of Medicine, Charles University, Prague, Czech Republic. ^31^Centro de Investigación Biomédica en Red de Enfermedades Hepàticas y Digestivas (Ciberehd), Hospital de Mataró, Consorci Sanitari del Maresme, Mataró, Spain. ^32^Service de Pneumologie, Hopital Bichat, APHP, Paris, France. ^33^Clinical Immunology Unit, Pediatric Infectious Disease Department, Faculty of Medicine and Pharmacy, Averroes University Hospital, LICIA Laboratoire d'immunologie clinique, d'inflammation et d'allergie, Hassann Ii University, Casablanca, Morocco. ^34^Endocrinology Unit, APHP Hôpitaux Universitaires Paris-Sud, Le Kremlin-Bicêtre, France. ^35^Department of Children's Diseases and Pediatric Surgery, I.Horbachevsky Ternopil National Medical University, Ternopil, Ukraine. ^36^Human Genome and Stem-Cell Research Center, University of São Paulo, São Paulo, Brazil. ^37^Hospital Insular, Las Palmas de Gran Canaria, Spain. ^38^Division of Critical Care Medicine, Department of Anesthesiology and Reanimation, Konya State Hospital, Konya, Turkey. ^39^MS Center, Spedali Civili, Brescia, Italy. ^40^Fondazione IRCCS Ca' Granda Ospedale Maggiore Policlinico, Milan, Italy. ^41^Bellvitge University Hospital, L'Hospitalet de Llobregat, Barcelona, Spain. ^42^Hopital Robert Debré, Paris, France. ^43^Pediatric Immuno-hematology Unit, Necker Enfants Malades Hospital, AP-HP, Paris, France. ^44^Department of Infectious and Tropical Diseases, University of Brescia, ASST Spedali Civili di Brescia, Brescia, Italy. ^45^Doctoral Health Care Center, Canarian Health System, Las Palmas de Gran Canaria, Spain. ^46^Hôpital Foch, Suresnes, France. ^47^Necker Hospital for Sick Children, Paris University, AP-HP, Paris, France. ^48^Pasteur Institute, Paris, France. ^49^McGill University Health Centre, Montreal, Canada. ^50^University Hospital and Research Institute “Germans Trias i Pujol”, IrsiCaixa AIDS Research Institute, UVic-UCC, Badalona, Spain. ^51^Clinical Biochemistry, Pathology, Paediatric Neurology and Molecular Medicine Departments and Biobank, Institut de Recerca Sant Joan de Déu and CIBERER-ISCIII, Esplugues, Spain. ^52^Division of Clinical Immunology and Allergy, Department of Internal Medicine, Necmettin Erbakan University, Meram Medical Faculty, Konya, Turkey. ^53^Department of Infectious Diseases and Clinical Microbiology, Konya Training and Research Hospital, Konya, Turkey. ^54^Hospital Universitari Vall d’Hebron, Barcelona, Spain. ^55^Pitié-Salpêtrière Hospital, Paris, France. ^56^Respiratory Diseases Division, IRCCS Policlinico San Matteo Foundation and University of Pavia, Pavia, Italy. ^57^Fundació Docència i Recerca Mútua Terrassa, Barcelona, Spain. ^58^UNSW Medicine, St Vincent's Clinical School; Department of Thoracic Medicine, St Vincent's Hospital Darlinghurst, Sidney, Australia. ^59^CHU Saint-Pierre, Université Libre de Bruxelles (ULB), Brussels, Belgium. ^60^Pediatric Intensive Care Unit, Robert-Debré University Hospital, APHP, Paris, France. ^61^Sorbonne Paris Nord, Hôpital Jean Verdier, APHP, Bondy, France. ^62^Specialized Immunology Laboratory of Dr. Shahrooei, Sina Medical Complex, Ahvaz, Iran. ^63^Centre de génétique humaine, CHU Besançon, Besançon, France. ^64^Sorbonne Université Médecine and APHP Sorbonne Université site Pitié-Salpêtrière, Paris, France. ^65^Department of Internal Medicine, Fondazione IRCCS Policlinico San Matteo, University of Pavia, Pavia, Italy. ^66^Intensive Care Unit, Georges Pompidou Hospital, APHP, Paris, France. ^67^Department of Pneumology, AZ Delta, Roeselare, Belgium. ^68^Institut Jérôme Lejeune, Paris, France. ^69^Department of Microbiology and Immunology, Faculty of Medicine, Mansoura University, Mansoura, Egypt. ^70^Department of Chest, Faculty of Medicine, Mansoura University, Mansoura, Egypt. ^71^Department of Medical Biochemistry and Molecular Biology, Faculty of Medicine, Mansoura University, Mansoura, Egypt. ^72^Faculty of Medicine, Division of Pediatric Infectious Diseases, Selcuk University, Konya, Turkey. ^73^Division of Pediatric Infectious Diseases, Ondokuz Mayıs University, Samsun, Turkey. ^74^Necmettin Erbakan University, Meram Medical Faculty, Division of Pediatric Allergy and Immunology, Konya, Turkey. ^75^Centre Hospitalier Fleyriat, Bourg-en-Bresse, France. ^76^Division of Clinical Immunology and Allergy, Department of Internal Medicine, Necmettin Erbakan University, Meram Medical Faculty, Konya, Turkey. ^77^Centre de Génétique, CHU Dijon, Dijon, France. ^78^APHP Tenon Hospital, Paris, France. ^79^Sorbonne Universités, UPMC University of Paris, Paris, France. ^80^Department of Clinical Immunology, Hospital Clínico San Carlos, Madrid, Spain. ^81^Genomics Division, Instituto Tecnológico y de Energías Renovables (ITER), Santa Cruz de Tenerife, Spain; CIBER de Enfermedades Respiratorias, Instituto de Salud Carlos III, Madrid, Spain; Research Unit, Hospital Universitario N.S. de Candelaria, Santa Cruz de Tenerife, Spain; Instituto de Tecnologías Biomédicas (ITB), Universidad de La Laguna, San Cristóbal de La Laguna, Spain. ^82^CHU Limoges and Inserm CIC 1435 & UMR 1092, Limoges, France. ^83^Infectious Diseases Unit, Department of Pediatrics, Hospital Sant Joan de Déu, Barcelona, Spain; Institut de Recerca Sant Joan de Déu, Spain; Universitat de Barcelona (UB), Barcelona, Spain. ^84^Institute of Genetics and Biophysics ‘Adriano Buzzati-Traverso’, IGB-CNR, Naples, Italy. ^85^Laboratory of Immunogenetics of Human Diseases, IdiPAZ Institute for Health Research, La Paz Hospital, Madrid, Spain. ^86^Hematology, APHP, Hopital Européen Georges Pompidou and Inserm UMR-S1140, Paris, France. ^87^Hospital General Universitario and Instituto de Investigación Sanitaria "Gregorio Marañón", Madrid, Spain. ^88^Bégin military Hospital, Bégin, France. ^89^Pediatric Intensive Care Unit, Hospital Sant Joan de Déu, Barcelona, Spain. ^90^Department of Internal Medicine, Hôpital Erasme, Université Libre de Bruxelles, Brussels, Belgium. ^91^Division of Critical Care Medicine, Department of Anesthesiology and Reanimation, Necmettin Erbakan University, Meram Medical Faculty, Konya, Turkey. ^92^Genomics Division, Instituto Tecnológico y de Energías Renovables (ITER), Santa Cruz de Tenerife, Spain. ^93^Assistance Publique Hôpitaux de Paris, Paris, France. ^94^Division of Allergy and Immunology, Necmettin Erbakan University, Meram Medical Faculty, Konya, Turkey. ^95^CNAG-CRG, Centre for Genomic Regulation (CRG), Barcelona Institute of Science and Technology (BIST); Universitat Pompeu Fabra (UPF), Barcelona, Spain. ^96^Department of Internal Medicine, National Reference Center for Rare Systemic Autoimmune Diseases, AP-HP, APHP-CUP, Hôpital Cochin, Paris, France. ^97^Ghent University Hospital, Ghent, Belgium. ^98^Sharjah Institute of Medical Research, College of Medicine, University of Sharjah, Sharjah, United Arab Emirates. ^99^Department of Biosciences and Nutrition, SE14183, Huddinge, Karolinska Institutet, Stockholm, Sweden. ^100^Pediatric Infectious Diseases Unit, Bakirkoy Dr. Sadi Konuk Training and Research Hospital, University of Health Sciences, Istanbul, Turkey. ^101^Department of Immunology, Hospital Universitario de Gran Canaria Dr. Negrín, Canarian Health System, Las Palmas de Gran Canaria, Spain. ^102^Intensive Care Unit, Marqués de Valdecilla Hospital, Santander, Spain. ^103^Hospital del Mar, Parc de Salut Mar, Barcelona, Spain. ^104^Intensive Care Unit, APHM, Marseille, France. ^105^CHU Lille, Lille, France. ^106^Department of Pediatrics, Columbia University, New York, NY, USA. ^107^Centre Hospitalier Intercommunal Poissy Saint Germain en Laye, Poissy, France. ^108^Fundació Docència i Recerca Mútua Terrassa, Barcelona, Spain. ^109^Hospital Sant Joan de Déu, Kids Corona Platfform, Barcelona, Spain. ^110^Selcuk University, Faculty of Medicine, Chest Diseases Department, Konya, Turkey. ^111^Division of Allergy and Immunology, Balikesir Ataturk City Hospital, Balikesir, Turkey. ^112^Division of Critical Care Medicine, Selcuk University, Faculty of Medicine, Konya, Turkey. ^113^Division of Pediatric Infectious Diseases, Prof. Dr. Cemil Tascıoglu City Hospital, Istanbul, Turkey. ^114^Departments of Infectious Diseases and Clinical Microbiology, Bakirkoy Dr. Sadi Konuk Training and Research Hospital, University of Health Sciences, Istanbul, Turkey. ^115^Meram Medical Faculty, Necmettin Erbakan University, Meram Medical Faculty, Konya, Turkey. ^116^Health Sciences University, Umraniye Education and Research Hospital, Istanbul, Turkey. ^117^Department of Immunology, 2nd Faculty of Medicine, Charles University and University Hospital in Motol, Prague, Czech Republic. ^118^Central Clinical Hospital of Ministry of the Interior and Administration in Warsaw, Warsaw, Poland. ^119^Oncobiologie Génétique Bioinformatique, PC Bio, CHU Besançon, Besançon, France. ^120^Paediatric Infectious Disease Unit, Hospital Authority Infectious Disease Center, Princess Margaret Hospital, Hong Kong (Special Administrative Region), China. ^121^Aix Marseille Univ, IRD, MEPHI, IHU Méditerranée Infection, Marseille, France. ^122^Department of Paediatrics and Adolescent Medicine, The University of Hong Kong, Hong Kong, China. ^123^National Centre for Infectious Diseases, Singapore. ^124^Hospital Universitario Reina Sofía, Cordoba, Spain. ^125^Imperial College, London, UK. ^126^Endocrinology and diabetes for children, AP-HP, Bicêtre Paris-Saclay Hospital, Le Kremlin-Bicêtre, France. ^127^Neurology Unit, APHP Pitié-Salpêtrière Hospital, Paris University, Paris, France. ^128^Intensive Care Unit, APHP Pitié-Salpêtrière Hospital, Paris University, Paris, France. ^129^National Centre for Infectious Diseases; Tan Tock Seng Hospital; Yong Loo Lin School of Medicine; Lee Kong Chian School of Medicine, Singapore. ^130^Department of Clinical Immunology and Infectious Diseases, National Research Institute of Tuberculosis and Lung Diseases, Shahid Beheshti University of Medical Sciences, Tehran, Iran. ^131^Clinical Tuberculosis and Epidemiology Research Center, National Research Institute of Tuberculosis and Lung Diseases (NRITLD), Shahid Beheshti University of Medical Sciences, Tehran, Iran. ^132^Hospital Sant Joan de Déu and University of Barcelona, Barcelona, Spain. ^133^Pediatric Infectious Diseases and Immunodeficiencies Unit, Hospital Universitari Vall d’Hebron, Vall d'Hebron Research Institute, Vall d’Hebron Barcelona Hospital Campus, Universitat Autònoma de Barcelona (UAB), Barcelona, Spain. ^134^Hospital Universitari Mutua de Terrassa, Universitat de Barcelona, Barcelona, Spain. ^135^IrsiCaixa AIDS Research Institute, ICREA, UVic-UCC, Research Institute “Germans Trias i Pujol”, Badalona, Spain. ^136^Department of Laboratory, Cruces University Hospital, Barakaldo, Bizkaia, Spain. ^137^University of New South Wales, Darlinghurst, NSW, Australia. ^138^APHP Pitié-Salpêtrière Hospital, Paris, France. ^139^Aix-Marseille University, APHM, Marseille, France. ^140^Robert Debré Hospital, Paris, France. ^141^APHP Cohin Hospital, Paris, France. ^142^Necmettin Erbakan University Meram Faculty of Medicine Department of Pediatric Infectious Diseases, Konya, Turkey. ^143^University Hospitals Leuven, Leuven, Belgium. ^144^Hospices Civils de Lyon, Hôpital de la Croix-Rousse, Lyon, France. ^145^Hôpital Erasme, Brussels, Belgium. ^146^CH Gonesse, Gonesse, France. ^147^Vascular Medicine, Georges Pompidou Hospital, APHP, Paris, France. ^148^Division of Pulmonary and Critical Care, University of Miami, Miami, FL, USA. ^149^Guanarteme Health Care Center, Canarian Health System, Las Palmas de Gran Canaria, Spain. ^150^Regional University Hospital of Málaga, Málaga, Spain. ^151^Aix-Marseille Université, Marseille, France. ^152^Department of General Paediatrics, Hôpital Bicêtre, AP-HP, University of Paris Saclay, Le Kremlin-Bicêtre, France. ^153^CHU de La Timone, Marseille, France. ^154^Centro Hospitalar Universitário de Lisboa Central, Lisbon, Portugal. ^155^Infectious Diseases Horizontal Technlogy Centre, A*STAR; Singapore Immunology Network, A*STAR, Singapore. ^156^Department of Pediatrics, Complejo Hospitalario Universitario Insular-Materno Infantil, Canarian Health System, Las Palmas de Gran Canaria, Spain. ^157^Regional University Hospital of Málaga, Málaga, Spain. ^158^Hospital Universitario Marqués de Valdecilla, Santander, Spain. ^159^Ataturk University Medical Faculty, Erzurum, Turkey. ^160^Bilkent University, Department of Molecular Biology and Genetics, Ankara, Turkey. ^161^Department of Laboratory Medicine, Karolinska Institutet, SE14186, Stockholm, Sweden. ^162^L'Hôpital Foch, Suresnes, France. ^163^Department of Immunology, Hospital Universitario 12 de Octubre, Instituto de Investigación Sanitaria Hospital 12 de Octubre (imas12), Madrid, Spain. ^164^APHP Hôpitaux Universitaires Paris-Sud, Le Kremlin-Bicêtre, France. ^165^Neurometabolic Diseases Laboratory, IDIBELL-Hospital Duran i Reynals, Barcelona; CIBERER U759, ISCiii, Madrid, Spain. ^166^Hospices Civils de Lyon, Lyon, France. ^167^Université de Lille, Inserm U1285, CHU Lille, Paris, France. ^168^Department of General Pediatrics, University Hospital Robert Debré, APHP, Paris, France. ^169^Necmettin Erbakan University, Konya, Turkey. ^170^Germans Trias i Pujol Hospital, Badalona, Spain. ^171^Medical Intensive Care Unit, Hopital de la Croix-Rousse, Hospices Civils de Lyon, Lyon, France. ^172^Pediatric Infectious Diseases and Immunodeficiencies Unit, Hospital Universitari Vall d’Hebron, Vall d'Hebron Research Institute, Vall d’Hebron Barcelona Hospital Campus, Barcelona, Spain. ^173^Department of Immunology, Hospital Universitario de Gran Canaria Dr. Negrín, Canarian Health System, Las Palmas de Gran Canaria, Spain; University Fernando Pessoa Canarias, Las Palmas de Gran Canaria, Spain. ^174^Neurometabolic Diseases Laboratory, IDIBELL-Hospital Duran i Reynals, Barcelona, Spain. ^175^Consorcio Hospital General Universitario, Valencia, Spain. ^176^APHP Hôpitaux Universitaires Paris-Sud, Paris, France. ^177^Virology Unit, Université de Paris, Cohin Hospital, APHP, Paris, France. ^178^Hospital San Pedro, Logroño, Spain. ^179^Respiratory medicine, Georges Pompidou Hospital, APHP, Paris, France. ^180^Department of Immunology, Hospital Clínico San Carlos, Madrid, Spain. ^181^Service de Médecine Intensive Réanimation, Institut de Cardiologie, Hopital Pitié-Salpêtrière, Paris, France. ^182^CHRU de Nancy, Hôpital d'Enfants, Vandoeuvre, France. ^183^Chair of Nephrology, University of Brescia, Brescia, Italy. ^184^Department of Immunology, 2nd Faculty of Medicine, Charles University and Motol University Hospital, Prague, Czech Republic. ^185^Clínica Universidad de Navarra, Madrid, Spain. ^186^HUS Helsinki University Hospital, Children and Adolescents, Rare Disease Center, and Inflammation Center, Adult Immunodeficiency Unit, Majakka, Helsinki, Finland. ^187^Fundació Docència i Recerca Mútua Terrassa, Terrassa, Spain. ^188^Hopital Européen Georges Pompidou, Paris, France. ^189^Department of Pediatrics, Faculty of Medicine, Mansoura University, Mansoura, Egypt. ^190^Critical Care Unit, Hospital Universitario de Gran Canaria Dr. Negrín, Canarian Health System, Las Palmas de Gran Canaria, Spain. ^191^CHU de Saint Etienne, Saint-Priest-en-Jarez, France. ^192^Shupyk National Medical Academy for Postgraduate Education, Kiev, Ukraine. ^193^Gustave Roussy Cancer Campus, Villejuif, France. ^194^Intensive Care Unit, Avicenne Hospital, APHP, Bobigny, France. ^195^Laboratory of Immunology and Histocompatibility, Saint-Louis Hospital, Paris University, Paris, France. ^196^Department of Internal Diseases and Pediatrics, Primary Immune Deficiency Research Laboratory, Centre for Primary Immunodeficiency Ghent, Jeffrey Modell Diagnosis and Research Centre, Ghent University Hospital, Ghent, Belgium. ^197^Department of Internal Medicine, Université de Paris, INSERM, U970, PARCC, F-75015, Paris, France. ^198^First Division of Anesthesiology and Critical Care Medicine, University of Brescia, ASST Spedali Civili di Brescia, Brescia, Italy. ^199^Intensive Care Department, Hospital Universitari MutuaTerrassa, Universitat Barcelona, Terrassa, Spain. ^200^Hospices Civils de Lyon, Lyon Sud Hospital, Lyon, France. ^201^Infanta Leonor University Hospital, Madrid, Spain. ^202^Hematology Department, ASST Spedali Civili di Brescia, Brescia, Italy. ^203^Pneumologie, Hôpital Avicenne, APHP, INSERM U1272, Université Sorbonne Paris Nord, Bobigny, France. ^204^Dermatology Unit, Laboratoire GAD, INSERM UMR1231 LNC, Université de Bourgogne, Dijon, France. ^205^University Hospital of Burgos, Burgos, Spain. ^206^Center of Human Genetics, Hôpital Erasme, Université Libre de Bruxelles, Brussels, Belgium. ^207^Department of Chest Diseases, Necmettin Erbakan University, Meram Medical Faculty, Konya, Turkey. ^208^CHU de Caen, Caen, France. ^209^Sorbonne Université, Service de Médecine Intensive Réanimation, Hôpital Tenon, Assistance Publique-Hôpitaux de Paris, Paris, France. ^210^General Intensive Care Unit, Konya Training and Research Hospital, Konya, Turkey. ^211^CHU de Nancy, Nancy, France. ^212^University of Lyon, CIRI, INSERM U1111, National Referee Centre RAISE, Pediatric Rheumatology, HFME, Hospices Civils de Lyon, Lyon, France. *Leader of the COVID-Clinicians group.

## COVID-STORM Clinicians

Giuseppe Foti^1^, Giacomo Bellani^1^, Giuseppe Citerio^1^, Ernesto Contro^1^, Alberto Pesci^2^, Maria Grazia Valsecchi^3^, Marina Cazzaniga^4^

^1^Department of Emergency, Anesthesia and Intensive Care, School of Medicine and Surgery, University of Milano-Bicocca, San Gerardo Hospital, Monza, Italy. ^2^Department of Pneumology, School of Medicine and Surgery, University of Milano-Bicocca, San Gerardo Hospital, Monza, Italy. ^3^Center of Bioinformatics and Biostatistics, School of Medicine and Surgery, University of Milano-Bicocca, San Gerardo Hospital, Monza, Italy. ^4^Phase I Research Center, School of Medicine and Surgery, University of Milano-Bicocca, San Gerardo Hospital, Monza, Italy.

## Imagine COVID Group

Christine Bole-Feysot^1^, Stanislas Lyonnet^1^*, Cécile Masson^1^, Patrick Nitschke^1^, Aurore Pouliet^1^, Yoann Schmitt^1^, Frederic Tores^1^, Mohammed Zarhrate^1^

^1^Imagine Institute, Université de Paris, INSERM UMR 1163, Paris, France. *Leader of the Imagine COVID Group.

## French COVID Cohort Study Group

Laurent Abel^1^, Claire Andrejak^2^, François Angoulvant^3^, Delphine Bachelet^4^, Romain Basmaci^5^, Sylvie Behillil^6^, Marine Beluze^7^, Dehbia Benkerrou^8^, Krishna Bhavsar^4^, François Bompart^9^, Lila Bouadma^4^, Maude Bouscambert^10^, Mireille Caralp^11^, Minerva Cervantes-Gonzalez^12^, Anissa Chair^4^, Alexandra Coelho^13^, Camille Couffignal^4^, Sandrine Couffin-Cadiergues^14^, Eric D’ortenzio^12^, Charlene Da Silveira^4^, Marie-Pierre Debray^4^, Dominique Deplanque^15^, Diane Descamps^16^, Mathilde Desvallées^17^, Alpha Diallo^18^, Alphonsine Diouf^13^, Céline Dorival^8^, François Dubos^19^, Xavier Duval^4^, Philippine Eloy^4^, Vincent V. E. Enouf^20^, Hélène Esperou^21^, Marina Esposito-Farese^4^, Manuel Etienne^22^, Nadia Ettalhaoui^4^, Nathalie Gault^4^, Alexandre Gaymard^10^, Jade Ghosn^4^, Tristan Gigante^23^, Isabelle Gorenne^4^, Jérémie Guedj^24^, Alexandre Hoctin^13^, Isabelle Hoffmann^4^, Salma Jaafoura^21^, Ouifiya Kafif^4^, Florentia Kaguelidou^25^, Sabina Kali^4^, Antoine Khalil^4^, Coralie Khan^17^, Cédric Laouénan^4^, Samira Laribi^4^, Minh Le^4^, Quentin Le Hingrat^4^, Soizic Le Mestre^18^, Hervé Le Nagard^24^, François-Xavier Lescure^4^, Yves Lévy^26^, Claire Levy-Marchal^27^, Bruno Lina^10^, Guillaume Lingas^24^, Jean Christophe Lucet^4^, Denis Malvy^28^, Marina Mambert^13^, France Mentré^4^, Noémie Mercier^18^, Amina Meziane^8^, Hugo Mouquet^20^, Jimmy Mullaert^4^, Nadège Neant^24^, Marion Noret^29^, Justine Pages^30^, Aurélie Papadopoulos^21^, Christelle Paul^18^, Nathan Peiffer-Smadja^4^, Ventzislava Petrov-Sanchez^18^, Gilles Peytavin^4^, Olivier Picone^31^, Oriane Puéchal^12^, Manuel Rosa-Calatrava^10^, Bénédicte Rossignol^23^, Patrick Rossignol^32^, Carine Roy^4^, Marion Schneider^4^, Caroline Semaille^12^, Nassima Si Mohammed^4^, Lysa Tagherset^4^, Coralie Tardivon^4^, Marie-Capucine Tellier^4^, François Téoulé^8^, Olivier Terrier^10^, Jean-François Timsit^4^, Théo Treoux^4^, Christelle Tual^33^, Sarah Tubiana^4^, Sylvie van der Werf^34^, Noémie Vanel^35^, Aurélie Veislinger^33^, Benoit Visseaux^16^, Aurélie Wiedemann^26^, Yazdan Yazdanpanah^36^

^1^Inserm UMR 1163, Paris, France. ^2^CHU Amiens, Amiens, France. ^3^Hôpital Necker, Paris, France. ^4^Hôpital Bichat, Paris, France. ^5^Hôpital Louis Mourrier, Colombes, France. ^6^Institut Pasteur, Paris, France. ^7^F-CRIN Partners Platform, AP-HP, Université de Paris, Paris, France. ^8^Inserm UMR 1136, Paris, France. ^9^Drugs for Neglected Diseases Initiative, Geneva, Switzerland. ^10^Inserm UMR 1111, Lyon, France. ^11^Inserm Transfert, Paris, France. ^12^REACTing, Paris, France. ^13^Inserm UMR 1018, Paris, France. ^14^Inserm, Pôle Recherche Clinique, France. ^15^CIC 1403 Inserm-CHU Lille, Paris, France. ^16^Université de Paris, IAME, INSERM UMR 1137, AP-HP, University Hospital Bichat Claude Bernard, Virology, F-75018 Paris, France. ^17^Inserm UMR 1219, Bordeaux, France. ^18^ANRS, Paris, France. ^19^CHU Lille, Lille, France. ^20^Pasteur Institute, Paris, France. ^21^Inserm sponsor, Paris, France. ^22^Rouen - SMIT, France. ^23^FCRIN INI-CRCT, Nancy, France. ^24^Inserm UMR 1137, Paris, France. ^25^Centre d'Investigation Clinique, Inserm CIC1426, Hôpital Robert Debré, Paris, France. ^26^Inserm UMR 955, Créteil, France; Vaccine Research Instiute (VRI), Paris, France. ^27^F-CRIN INI-CRCT, Paris, France. ^28^Bordeaux - SMIT, France. ^29^RENARCI, Annecy, France. ^30^Hôpital Robert Debré, Paris, France. ^31^Colombes - Louis Mourier - Gynécologie, France. ^32^University of Lorraine, Plurithematic Clinical Investigation Centre Inserm CIC-P; 1433, Inserm U1116, CHRU Nancy Hopitaux de Brabois, F-CRIN INI-CRCT; (Cardiovascular and Renal Clinical Trialists), Nancy, France. ^33^Inserm CIC-1414, Rennes, France. ^34^Institut Pasteur, UMR 3569 CNRS, Université de Paris, Paris, France. ^35^Hôpital la timone, Marseille, France. ^36^Paris - Bichat - SMIT, France.

## The Milieu Intérieur Consortium

Laurent Abel^1^, Andres Alcover^2^, Hugues Aschard^2^, Kalla Astrom^3^, Philippe Bousso^2^, Pierre Bruhns^2^, Ana Cumano^2^, Caroline Demangel^2^, Ludovic Deriano^2^, James Di Santo^2^, Françoise Dromer^2^, Gérard Eberl^2^, Jost Enninga^2^, Jacques Fellay^4^, Ivo Gomperts-Boneca^2^, Milena Hasan^2^, Serge Hercberg^5^, Olivier Lantz^6^, Hugo Mouquet^2^, Etienne Patin^2^, Sandra Pellegrini^2^, Stanislas Pol^7^, Antonio Rausell^8^, Lars Rogge^2^, Anavaj Sakuntabhai^2^, Olivier Schwartz^2^, Benno Schwikowski^2^, Spencer Shorte^2^, Frédéric Tangy^2^, Antoine Toubert^9^, Mathilde Touvier^10^, Marie-Noëlle Ungeheuer^2^, Matthew L. Albert^11^*, Darragh Duffy^2^*, Lluis Quintana-Murci^2^*

^1^INSERM U1163, University of Paris, Imagine Institute, Paris, France. ^2^Pasteur Institute, Paris, France. ^3^Lund University, Lund, Sweden. ^4^EPFL, Lausanne, Switzerland. ^5^Université Paris 13, Paris, France. ^6^Curie Institute, Paris, France. ^7^Cochin Hospital, Paris, France. ^8^INSERM UMR 1163 – Institut Imagine, Paris, France. ^9^Hôpital Saint-Louis, Paris, France. ^10^Sorbonne Paris Nord University, Inserm U1153, Inrae U1125, Cnam, Nutritional Epidemiology Research Team (EREN), Bobigny, France. ^11^In Sitro, San Francisco, CA, USA. *Co-coordinators of The Milieu Intérieur Consortium. Additional information can be found at: www.milieuinterieur.fr/en.

## CoV-Contact Cohort

Loubna Alavoine^1^, Karine K. A. Amat^2^, Sylvie Behillil^3^, Julia Bielicki^4^, Patricia Bruijning^5^, Charles Burdet^6^, Eric Caumes^7^, Charlotte Charpentier^8^, Bruno Coignard^9^, Yolande Costa^1^, Sandrine Couffin-Cadiergues^10^, Florence Damond^8^, Aline Dechanet^11^, Christelle Delmas^10^, Diane Descamps^8^, Xavier Duval^1^, Jean-Luc Ecobichon^1^, Vincent Enouf^3^, Hélène Espérou^10^, Wahiba Frezouls^1^, Nadhira Houhou^11^, Emila Ilic-Habensus^1^, Ouifiya Kafif^11^, John Kikoine^11^, Quentin Le Hingrat^8^, David Lebeaux^12^, Anne Leclercq^1^, Jonathan Lehacaut^1^, Sophie Letrou^1^, Bruno Lina^13^, Jean-Christophe Lucet^14^, Denis Malvy^15^, Pauline Manchon^11^, Milica Mandic^1^, Mohamed Meghadecha^16^, Justina Motiejunaite^17^, Mariama Nouroudine^1^, Valentine Piquard^11^, Andreea Postolache^11^, Caroline Quintin^1^, Jade Rexach^1^, Layidé Roufai^10^, Zaven Terzian^11^, Michael Thy^18^, Sarah Tubiana^1^, Sylvie van der Werf^3^, Valérie Vignali^1^, Benoit Visseaux^8^, Yazdan Yazdanpanah^14^

^1^Centre d'Investigation Clinique, Inserm CIC 1425, Hôpital Bichat Claude Bernard, APHP, Paris, France. ^2^IMEA Fondation Léon M'Ba, Paris, France. ^3^Institut Pasteur, UMR 3569 CNRS, Université de Paris, Paris, France. ^4^University of Basel Children’s Hospital, Basel, Switzerland. ^5^Julius Center for Health Sciences and Primary Care, University Medical Center Utrecht, Utrecht, Netherlands. ^6^Université de Paris, IAME, Inserm UMR 1137, F-75018, Paris, France; Hôpital Bichat Claude Bernard, APHP, Paris, France. ^7^Hôpital Pitiè Salpétriere, APHP, Paris, France. ^8^Université de Paris, IAME, INSERM UMR 1137, AP-HP, University Hospital Bichat Claude Bernard, Virology, F-75018 Paris, France. ^9^Santé Publique France, Saint Maurice, France. ^10^Pole Recherche Clinique, Inserm, Paris, France. ^11^Hôpital Bichat Claude Bernard, APHP, Paris, France. ^12^APHP, Paris, France. ^13^Virpath Laboratory, International Center of Research in Infectiology, Lyon University, INSERM U1111, CNRS UMR 5308, ENS, UCBL, Lyon, France . ^14^IAME Inserm UMR 1138, Hôpital Bichat Claude Bernard, APHP, Paris, France. ^15^Service des Maladies Infectieuses et Tropicales, Groupe Pellegrin, Place Amélie-Raba-Léon, Bordeaux, France. ^16^Hôpital Hotel Dieu, APHP, Paris, France. ^17^Service des explorations fonctionnelles, Hôpital Bichat - Claude Bernard, APHP, Paris, France. ^18^Center for Clinical Investigation, Assistance Publique-Hôpitaux de Paris, Bichat-Claude Bernard University Hospital, Paris, France.

## Amsterdam UMC Covid-19 Biobank

Michiel van Agtmael^1^, Anna Geke Algera^2^, Frank van Baarle^2^, Diane Bax^3^, Martijn Beudel^4^, Harm Jan Bogaard^5^, Marije Bomers^1^, Lieuwe Bos^2^, Michela Botta^2^, Justin de Brabander^6^, Godelieve Bree^6^, Matthijs C. Brouwer^4^, Sanne de Bruin^2^, Marianna Bugiani^7^, Esther Bulle^2^, Osoul Chouchane^1^, Alex Cloherty^3^, Paul Elbers^2^, Lucas Fleuren^2^, Suzanne Geerlings^1^, Bart Geerts^8^, Theo Geijtenbeek^9^, Armand Girbes^2^, Bram Goorhuis^1^, Martin P. Grobusch^1^, Florianne Hafkamp^9^, Laura Hagens^2^, Jorg Hamann^10^, Vanessa Harris^1^, Robert Hemke^11^, Sabine M. Hermans^1^, Leo Heunks^2^, Markus W. Hollmann^8^, Janneke Horn^2^, Joppe W. Hovius^1^, Menno D. de Jong^12^, Rutger Koning^4^, Niels van Mourik^2^, Jeaninne Nellen^1^, Frederique Paulus^2^, Edgar Peters^1^, Tom van der Poll^1^, Benedikt Preckel^8^, Jan M. Prins^1^, Jorinde Raasveld^2^, Tom Reijnders^1^, Michiel Schinkel^1^, Marcus J. Schultz^2^, Alex Schuurman^13^, Kim Sigaloff^1^, Marry Smit^2^, Cornelis S. Stijnis^1^, Willemke Stilma^2^, Charlotte Teunissen^14^, Patrick Thoral^2^, Anissa Tsonas^2^, Marc van der Valk^1^, Denise Veelo^8^, Alexander P. J. Vlaar^15^, Heder de Vries^2^, Michèle van Vugt^1^, W. Joost Wiersinga^1^, Dorien Wouters^16^, A. H. (Koos) Zwinderman^17^, Diederik van de Beek^18^*

^1^Department of Infectious Diseases, Amsterdam UMC, Amsterdam, Netherlands. ^2^Department of Intensive Care, Amsterdam UMC, Amsterdam, Netherlands. ^3^Experimental Immunology, Amsterdam UMC, Amsterdam, Netherlands. ^4^Department of Neurology, Amsterdam UMC, Amsterdam Neuroscience, Amsterdam, Netherlands. ^5^Department of Pulmonology, Amsterdam UMC, Amsterdam, Netherlands. ^6^Department of Infectious Diseases, Amsterdam UMC, Amsterdam, Netherlands. ^7^Department of Pathology, Amsterdam UMC, Amsterdam, Netherlands. ^8^Department of Anesthesiology, Amsterdam UMC, Amsterdam, Netherlands. ^9^Department of Experimental Immunology, Amsterdam UMC, Amsterdam, Netherlands. ^10^Amsterdam UMC, Netherlands Biobank Core Facility, Amsterdam UMC, Amsterdam, Netherlands. ^11^Department of Radiology, Amsterdam UMC, Amsterdam, Netherlands. ^12^Department of Medical Microbiology, Amsterdam UMC, Amsterdam, Netherlands. ^13^Department of Internal Medicine, Amsterdam UMC, Amsterdam, Netherlands. ^14^Neurochemical Laboratory, Amsterdam UMC, Amsterdam, Netherlands. ^15^Deparment of Intensive Care, Amsterdam UMC, Amsterdam, Netherlands. ^16^Department of Clinical Chemistry, Amsterdam UMC, Amsterdam, Netherlands. ^17^Department of Clinical Epidemiology, Biostatistics and Bioinformatics, Amsterdam UMC, Amsterdam, Netherlands. ^18^Department of Neurology, Amsterdam UMC, Amsterdam, Netherlands. *Leader of the AMC consortium.

## COVID Human Genetic Effort

Laurent Abel^1^, Alessandro Aiuti^2^, Saleh Al Muhsen^3^, Fahd Al-Mulla^4^, Mark S. Anderson^5^, Andrés Augusto Arias^6^, Hagit Baris Feldman^7^, Dusan Bogunovic^8^, Alexandre Bolze^9^, Anastasiia Bondarenko^10^, Ahmed A. Bousfiha^11^, Petter Brodin^12^, Yenan Bryceson^12^, Carlos D. Bustamante^13^, Manish Butte^14^, Giorgio Casari^15^, Samya Chakravorty^16^, John Christodoulou^17^, Elizabeth Cirulli^9^, Antonio Condino-Neto^18^, Megan A. Cooper^19^, Clifton L. Dalgard^20^, Joseph L. DeRisi^21^, Murkesh Desai^22^, Beth A. Drolet^23^, Sara Espinosa^24^, Jacques Fellay^25^, Carlos Flores^26^, Jose Luis Franco^27^, Peter K. Gregersen^28^, Filomeen Haerynck^29^, David Hagin^30^, Rabih Halwani​^31^, Jim Heath^32^, Sarah E. Henrickson^33^, Elena Hsieh^34^, Kohsuke Imai^35^, Yuval Itan^8^, Timokratis Karamitros^36^, Kai Kisand^37^, Cheng-Lung Ku^38^, Yu-Lung Lau^39^, Yun Ling^40^, Carrie L. Lucas^41^, Tom Maniatis^42^, Davoud Mansouri^43^, Laszlo Marodi^44^, Isabelle Meyts^45^, Joshua D. Milner^46^, Kristina Mironska^47^, Trine Mogensen^48^, Tomohiro Morio^49^, Lisa F. P. Ng^50^, Luigi D. Notarangelo^51^, Giuseppe Novelli^52^, Antonio Novelli^53^, Cliona O'Farrelly^54^, Satoshi Okada^55^, Tayfun Ozcelik^56^, Rebeca Perez de Diego^57^, Anna M. Planas^58^, Carolina Prando^59^, Aurora Pujol^60^, Lluis Quintana-Murci^61^, Laurent Renia^62^, Alessandra Renieri^63^, Carlos Rodríguez-Gallego^64^, Vanessa Sancho-Shimizu^65^, Vijay Sankaran^66^, Kelly Schiabor Barrett^9^, Mohammed Shahrooei^67^, Andrew Snow^68^, Pere Soler-Palacín^69^, András N. Spaan^70^, Stuart Tangye^71^, Stuart Turvey^72^, Furkan Uddin^73^, Mohammed J. Uddin^74^, Diederik van de Beek^75^, Sara E. Vazquez^76^, Donald C. Vinh^77^, Horst von Bernuth^78^, Nicole Washington^9^, Pawel Zawadzki^79^, Helen C. Su^51^*, Jean-Laurent Casanova^80^*

^1^INSERM U1163, University of Paris, Imagine Institute, Paris, France. ^2^San Raffaele Telethon Institute for Gene Therapy, IRCCS Ospedale San Raffaele, Milan, Italy. ^3^King Saud University, Riyadh, Saudi Arabia. ^4^Dasman Diabetes Institute, Department of Genetics and Bioinformatics, Dasman, Kuwait. ^5^University of California, San Francisco, San Francisco, CA, USA. ^6^Universidad de Antioquia, Group of Primary Immunodeficiencies, Antioquia, Colombia. ^7^The Genetics Institute, Tel Aviv Sourasky Medical Center and Sackler Faculty of Medicine, Tel Aviv University, Tel Aviv, Israel. ^8^Icahn School of Medicine at Mount Sinai, New York, NY, USA. ^9^Helix, San Mateo, CA, USA. ^10^Shupyk National Medical Academy for Postgraduate Education, Kiev, Ukraine. ^11^Clinical Immunology Unit, Pediatric Infectious Disease Department, Faculty of Medicine and Pharmacy, Averroes University Hospital, LICIA Laboratoire d'immunologie clinique, d'inflammation et d'allergie, Hassann Ii University, Casablanca, Morocco. ^12^Karolinska Institute, Stockholm, Sweden. ^13^Stanford University, Stanford, CA, USA. ^14^University of California, Los Angeles, CA, USA. ^15^Medical Genetics, IRCCS Ospedale San Raffaele, Milan, Italy. ^16^Department of Pediatrics and Children’s Healthcare of Atlanta, Emory University, Atlanta, GA, USA. ^17^Murdoch Children's Research Institute, Victoria, Australia. ^18^University of São Paulo, São Paulo, Brazil. ^19^Washington University School of Medicine, St. Louis, MO, USA. ^20^The American Genome Center; Uniformed Services University of the Health Sciences, Bethesda, MD, USA. ^21^University of California San Francisco; Chan Zuckerberg Biohub, San Francisco, CA, USA. ^22^Bai Jerbai Wadia Hospital for Children, Mumbai, India. ^23^ School of Medicine and Public Health, University of Wisconsin, Madison, WI, USA. ^24^Instituto Nacional de Pediatria (National Institute of Pediatrics), Mexico City, Mexico. ^25^Swiss Federal Institute of Technology Lausanne, Lausanne, Switzerland. ^26^Research Unit, Hospital Universitario Nuestra Señora de Candelaria, Canarian Health System, Santa Cruz de Tenerife, Spain. ^27^University of Antioquia, Medellín, Colombia. ^28^Feinstein Institute for Medical Research, Northwell Health USA, Manhasset, NY, USA. ^29^Department of Paediatric Immunology and Pulmonology, Centre for Primary Immunodeficiency Ghent (CPIG), PID Research Laboratory, Jeffrey Modell Diagnosis and Research Centre, Ghent University Hospital, Edegem, Belgium. ^30^The Genetics Institute Tel Aviv Sourasky Medical Center, Tel Aviv, Israel. ^31^Sharjah Institute of Medical Research, College of Medicine, University of Sharjah, Sharjah, United Arab Emirates. ^32^Institute for Systems Biology, Seattle, WA, USA. ^33^Children's Hospital of Philadelphia, Philadelphia, PA, USA. ^34^Anschutz Medical Campus, Aurora, CO, USA. ^35^Riken, Tokyo, Japan. ^36^Hellenic Pasteur Institute, Athens, Greece. ^37^University of Tartu, Tartu, Estonia. ^38^Chang Gung University, Taoyuan County, Taiwan. ^39^The University of Hong Kong, Hong Kong, China. ^40^Shanghai Public Health Clinical Center, Fudan University, Shanghai, China. ^41^Yale School of Medicine, New Haven, CT, USA. ^42^New York Genome Center, New York, NY, USA. ^43^Shahid Beheshti University of Medical Sciences, Tehran, Iran. ^44^Semmelweis University Budapest, Budapest, Hungary. ^45^KU Leuven, Department of Immunology, Microbiology and Transplantation, Leuven, Belgium. ^46^Columbia University Medical Center, New York, NY, USA. ^47^University Clinic for Children's Diseases, Skopje, North Macedonia. ^48^Aarhus University, Aarhus, Denmark. ^49^Tokyo Medical & Dental University Hospital, Tokyo, Japan. ^50^Singapore Immunology Network, Singapore. ^51^National Institute of Allergy and Infectious Diseases, National Institutes of Health, Bethesda, MD, USA. ^52^Department of Biomedicine and Prevention, University of Rome “Tor Vergata,” Rome, Italy. ^53^Bambino Gesù Children's Hospital, Rome, Italy. ^54^Trinity College, Dublin, Ireland. ^55^Hiroshima University, Hiroshima, Japan. ^56^Bilkent University, Ankara, Turkey. ^57^Laboratory of Immunogenetics of Human Diseases, Innate Immunity Group, IdiPAZ Institute for Health Research, La Paz Hospital, Madrid, Spain. ^58^IIBB-CSIC, IDIBAPS, Barcelona, Spain. ^59^Faculdades Pequeno Príncipe e Instituto de Pesquisa Pelé Pequeno Príncipe, Curitiba, Brazil. ^60^Neurometabolic Diseases Laboratory, IDIBELL - Hospital Duran I Reynals; Catalan Institution for Research and Advanced Studies (ICREA); CIBERER U759, ISCiii Madrid Spain, Barcelona, Spain. ^61^Institut Pasteur (CNRS UMR2000) and Collège de France, Paris, France. ^62^Infectious Diseases Horizontal Technology Center and Singapore Immunology Network, Agency for Science Technology (A*STAR), Singapore. ^63^Medical Genetics, University of Siena, Italy; Genetica Medica, Azienda Ospedaliero-Universitaria Senese, GEN-COVID Multicenter Study, Italy. ^64^Hospital Universitario de Gran Canaria Dr Negrín, Canarian Health System, Canary Islands, Spain. ^65^Imperial College London, London, UK. ^66^Boston Children's Hospital, Harvard Medical School, Boston, MA, USA. ^67^Saeed Pathobiology and Genetic Laboratory, Tehran, Iran. ^68^Uniformed Services University of the Health Sciences (USUHS), Bethesda, MD, USA. ^69^Hospital Universitari Vall d'Hebron, Barcelona, Spain. ^70^University Medical Center Utrecht, Amsterdam, Netherlands. ^71^Garvan Institute of Medical Research, Sydney, Australia. ^72^The University of British Columbia, Vancouver, Canada. ^73^Holy Family Red Crescent Medical College; Centre for Precision Therapeutics, NeuroGen Children's Healthcare; Genetics and Genomic Medicine Centre, NeuroGen Children's Healthcare, Dhaka, Bangladesh. ^74^Mohammed Bin Rashid University of Medicine and Health Sciences, College of Medicine, Dubai, United Arab Emirates; The Centre for Applied Genomics, Department of Genetics and Genome Biology, The Hospital for Sick Children, Toronto, Ontario, Canada. ^75^Amsterdam UMC, University of Amsterdam, Department of Neurology, Amsterdam Neuroscience, Amsterdam, Netherlands. ^76^University of California, San Francisco, San Francisco, CA, USA. ^77^McGill University Health Centre, Montreal, Canada. ^78^Charité - Berlin University Hospital Center, Berlin, Germany. ^79^Molecular Biophysics Division, Faculty of Physics, A. Mickiewicz University, Uniwersytetu Poznanskiego 2, Poznań, Poland. ^80^The Rockefeller University, Howard Hughes Medical Institute, Necker Hospital, New York, NY, USA. *Leaders of the COVID Human Genetic Effort.
